# National and state-level analysis of peripheral nerve stimulation reimbursement patterns

**DOI:** 10.1016/j.inpm.2026.100826

**Published:** 2026-10-21

**Authors:** Evan J. Mizerak, Jay Karri, David A. Provenzano, Ryan S. D'Souza

**Affiliations:** a Mayo Clinic Alix School of Medicine, Rochester, USA; b Departments of Orthopaedic Surgery and Anesthesiology, University of Maryland School of Medicine, Baltimore, MD, USA; c Pain Diagnostics and Interventional Care, Sewickley, PA, USA; d Department of Anesthesiology and Perioperative Medicine, Mayo Clinic, Rochester, MN, USA

**Keywords:** Neuromodulation, Medicaid, Medicare, Public health, Health disparities

## Abstract

**Background:**

Peripheral nerve stimulation (PNS) is an increasingly utilized neuromodulation therapy for refractory chronic pain. However, physician reimbursement patterns for PNS procedures under Medicare, Medicaid, and other insurers remain poorly characterized. Because Medicaid reimbursement influences provider participation and patient access – particularly among low-income populations – quantifying these differences may highlight barriers to equitable access to neuromodulation.

**Objective:**

To quantify interstate variation in Medicaid reimbursement on an absolute basis and relative to Medicare for common PNS procedures, and to analyze national Medicare utilization and payment trends for these procedures.

**Methods:**

State-level Medicaid professional fee reimbursement rates for three common PNS CPT codes (64555: percutaneous lead implantation; 64575: open lead implantation; 64590: pulse generator insertion/replacement) as well as CPT 64553 (percutaneous cranial nerve lead implantation) were compared with corresponding 2026 Medicare Physician Fee Schedule rates. Medicaid rates were extracted from publicly available 2026 state fee schedules and analyzed before and after Medicare Wage Index adjustment. Medicaid-to-Medicare Indices (MMI) and reimbursement differentials normalized by work relative value units (wRVUs) were calculated to characterize the size and consistency of this payer gap across states and procedures. Secondary analyses examined national Medicare utilization and payment trends (2013–2024) using the CMS Physician & Other Practitioners by Geography and Service dataset to assess whether reimbursement patterns aligned with changes in procedural demand over time.

**Results:**

Median wage-adjusted Medicaid reimbursement was less than Medicare for all four codes: 32.1% for CPT 64555 (facility setting), 27.3% for CPT 64555 (non-facility setting), 14.0% for CPT 64575 (facility), 19.2% for CPT 64590 (facility), 27.3% for CPT 64590 (non-facility), 26.8% for CPT 64553 (facility), and 36.7% for CPT 64553 (non-facility). Reimbursement fell below Medicare parity in 61.7% to 86.5% of evaluated states depending on code and setting. From 2013 to 2024, Medicare utilization trends diverged by procedure: services, providers, and beneficiaries increased substantially for CPT 64555 and CPT 64590, but decreased for CPT 64575 and CPT 64553, even as average standardized payment rose for all four codes.

**Conclusions:**

Medicaid reimbursement for common PNS procedures is consistently lower than Medicare, with largest shortfall and interstate variability differing meaningfully by procedure and site of service. These procedure-specific disparities, alongside divergent utilization trends, suggest that targeted reimbursement adjustments may be needed to mitigate access barriers for Medicaid beneficiaries.

## 1 Introduction

Peripheral nerve stimulation (PNS) is a minimally invasive neuromodulation technique that has been used to treat chronic pain for over 50 years, with percutaneously implanted PNS leads and neurostimulators receiving FDA clearance beginning in 2015 [1]. Consensus clinical guidelines support the use of implantable PNS across a range of conditions refractory to conservative management [2]. These include peripheral neuropathic pain, post-surgical pain syndromes, complex region pain syndrome, and other chronic pain etiologies [3], [4], [5]. A recent two-year multicenter analysis found sustained reductions in pain intensity and opioid consumption following both temporary and permanent PNS, and a systematic review and meta-analysis of analgesic outcomes reported continued benefit lasting for several years following implantation [6], [7], [8], [9]. As PNS adoption grows, driven by the proven durability and efficacy of the modality, understanding how it is reimbursed by public payers is increasingly relevant to patient access.

With PNS utilization continuing to rise, an increasing share of recipients are covered by fee-for-service Medicare, the nation's largest single payer of healthcare services. The prevalence of chronic pain rises substantially with age, affecting approximately one-third of older adults and contributing significantly to disability and healthcare utilization [6], [7]. Concurrently, the Medicare beneficiary population is projected to expand considerably over the coming decades, with total Medicare expenditures expected to approach two trillion dollars annually by the mid-2030s [10]. As healthcare spending continues to increase, understanding reimbursement patterns for emerging pain interventions has become increasingly important. Multiple investigations have demonstrated substantial inflation-adjusted declines in Medicare reimbursement for common surgical and interventional procedures despite rising practice costs [11], [12]. Some analyses have reported decreases exceeding 30% over the past two decades, with average annual reimbursement reductions approaching 2% [12].

However, reimbursement trends specific to PNS implantation procedures remain poorly characterized. Given the growing utilization of PNS for chronic pain management, evaluation of longitudinal reimbursement trends may provide important insight into the economic factors influencing adoption, accessibility, and long-term sustainability of this evolving neuromodulation therapy. As contemporary PNS technologies have only recently achieved widespread clinical adoption, reimbursement patterns may play a particularly important role in shaping utilization.

Because Medicaid is jointly financed and administered by the federal and state governments, states retain substantial discretion in determining reimbursement policies beyond federally mandated requirements. As a result, payment rates, coverage policies, and delivery structures vary considerably across state Medicaid programs, creating meaningful differences in provider reimbursement and patient access to care. Although Medicaid expenditures have risen steadily over the past decade and are projected to continue growing at an average annual rate of approximately 6% through 2033 [13], [14], reimbursement patterns remain highly heterogeneous. Prior studies have demonstrated significant interstate variation in Medicaid reimbursement for spine and pain-related procedures [15]^,^ [16], as well as for opioid-sparing chronic pain treatment [17]. Despite these findings, reimbursement trends specific to PNS procedures have not been thoroughly characterized. It remains unclear whether the interstate disparities documented for other pain procedures extend to PNS, and, if so, where access to this increasingly used therapy may be most impacted.

Accordingly, the principal objective of this study was to characterize variation in state Medicaid coverage and reimbursement for common Current Procedural Terminology (CPT) codes related to PNS therapy (64555, 64575, 64590) across individual states. Because no study, to our knowledge, has compared Medicaid and Medicare reimbursement for PNS services across states, we sought to quantify Medicaid reimbursement as a proportion of Medicare reimbursement for each CPT code and examine geographic variation in these relationships. Analysis of these reimbursement differentials may enhance understanding of interstate disparities in payment practices and identify potential economic barriers affecting access to PNS therapy. Based on prior studies demonstrating lower Medicaid reimbursement for interventional pain procedures [15], [16], [18], we hypothesized that Medicare reimbursement would exceed Medicaid reimbursement for each PNS-related procedure included in the analysis.

## 2 Methods

### 2.1 Procedural code selection

This study (IRB Application #: 25-014043) was deemed as exempt from full review by the Mayo Clinic Institutional Review Board (IRB). Three commonly used CPT codes related to the implantation and management of PNS systems were selected for reimbursement analysis (eTable 1): 64555 (percutaneous implantation of neurostimulator electrode array; peripheral nerve), 64575 (open implantation of neurostimulator electrode array; peripheral nerve), and 64590 (insertion or replacement of peripheral neurostimulator pulse generator or receiver). These codes were selected to represent the principal implantation-related components of PNS therapy, including both lead placement and pulse generator implantation, while also enabling comparison of reimbursement between percutaneous and open surgical approaches. Advances in PNS technology have led to widespread adoption of percutaneous lead placement techniques [3], making CPT 64555 representative of a key procedural component across contemporary PNS systems. Pulse generator implantation (CPT 64590) is generally understood to be the most substantial cost driver of neurostimulation device placement [19], making it particularly important for reimbursement analysis.

As a post-hoc extension, CPT 64553 (percutaneous implantation of neurostimulator electrode array; cranial nerve) was added to the analysis. This code was included because it captures cranial nerve branch neurostimulation procedures, which are anatomically distinct but procedurally analogous to peripheral lead implantation procedures [20], [21]. Inclusion of CPT 64553 permitted comparison of reimbursement patterns between clinically related procedures that may involve different physician specialties and RVU weighting.

### 2.2 Data sources and data collection

The publicly available Centers for Medicare & Medicaid Services (CMS) Physician Fee Schedule (PFS) tool for the year 2026 was used to obtain Medicare fee-for-service rates [22]. Fee schedule amounts in the PFS incorporate geographic variation in practice costs by including a geographic practice cost index for work, practice expense, and malpractice relative value units (RVUs). The average physician professional fee reimbursement rates for a facility across all Medicare Administrative Contractor (MAC) localities was used for multi-locality states due the lack of publicly available population estimates by MAC locality to be used for weighting by population.

Medicaid reimbursement rates for each state were obtained from physician fee schedules available on state Medicaid program websites between April 1, 2026 and June 30, 2026. For CPT 64555, CPT 64590, and CPT 64553, which may be billed in either facility or non-facility (office) settings, the physician professional fee was extracted separately for each site of service when available, excluding any modifier codes. For CPT 64575, which is performed exclusively in facility settings, only the facility professional fee was extracted. Rarely, a state was excluded if its fee schedule was not publicly available or its Medicaid program was administered through managed care organizations without a single statewide fee schedule. Attempts at verifying ambiguous or unclear rates were made by contacting the state Medicaid office via email. If no response was received, state offices were contacted again within a 2-week timeframe. The data extraction process for Medicaid reimbursement rates is summarized in Fig. 1.

**Fig. 1:**
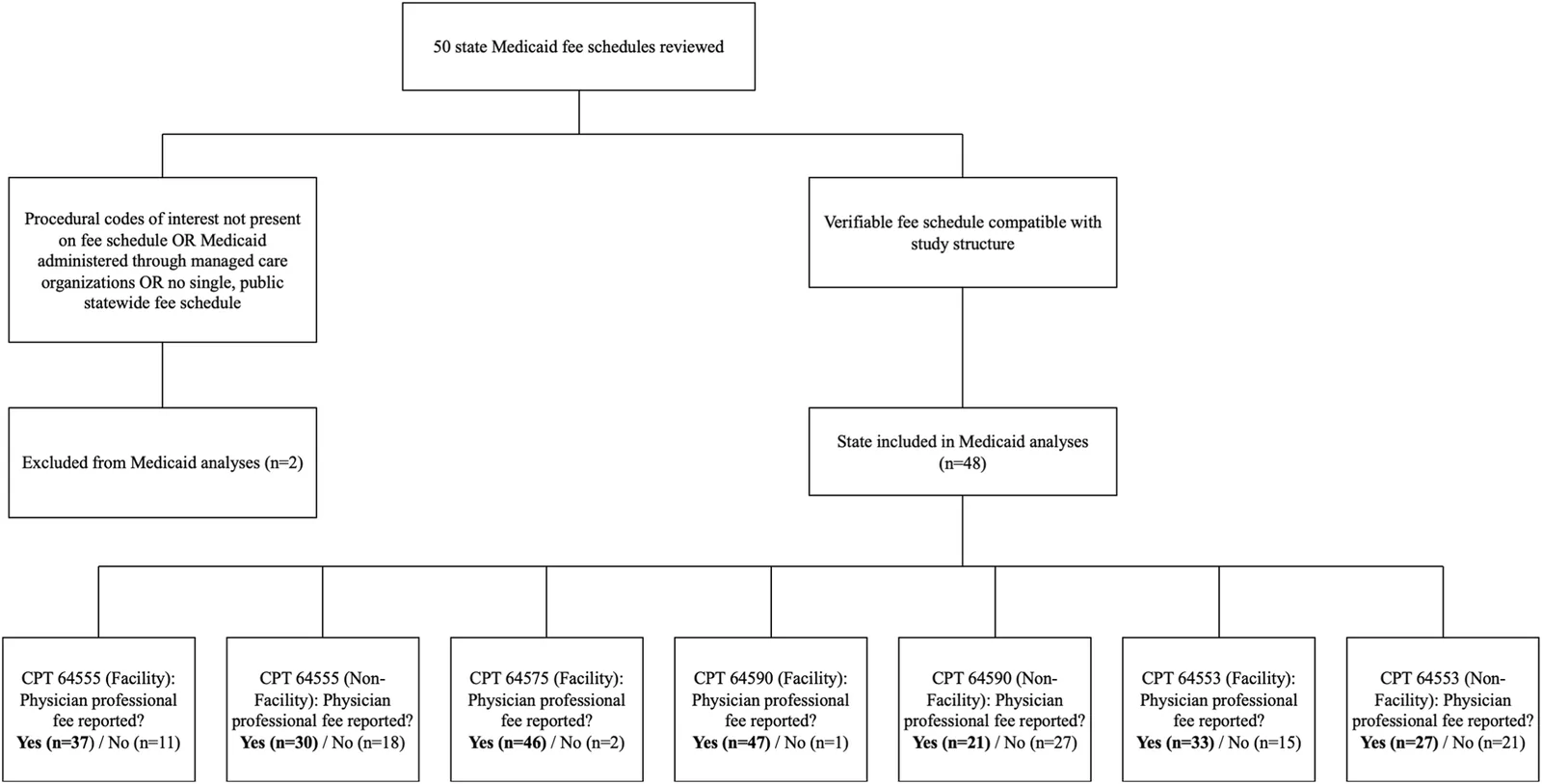
Selection and Extraction of Medicaid State DataSchematic conveying the Medicaid data selection and extraction process. Reimbursement rates were extracted from publicly available fee schedules between April 1, 2026, and June 30, 2026.

Values for Medicare Wage Index (MWI) and work Relative Value Units (wRVUs) were extracted from the CMS website for the year 2026 [22]. To account for regional variation in labor expenses, we incorporated the MWI, a geographic adjustment factor used by CMS within several Medicare payment systems. An MWI value of 1.00 corresponds to the national average labor cost, whereas values above and below 1.00 indicate labor costs that are respectively higher or lower than the national average. Wage-adjusted Medicaid reimbursement was calculated by applying the CMS-designated MWI for each state to the corresponding publicly reported reimbursement amount [23]. This approach is consistent with methodologies previously described by Lalezari et al. [18] and subsequently employed in analyses of spine procedure reimbursements by Henderson et al. [16] and Casper et al. [15].

To further standardize reimbursement comparisons, wRVUs were incorporated into the analysis. Within the CMS Resource-Based Relative Value Scale (RBRVS), wRVUs quantify the physician work associated with a given service, including factors such as procedural complexity, technical skill, clinical judgment, and time required for completion. Procedures demanding greater physician effort are therefore assigned higher wRVU values. To evaluate differences in reimbursement independent of procedural workload, the absolute reimbursement differential between Medicaid and Medicare was normalized by the corresponding CPT code's wRVU allocation. This yielded a reimbursement difference per unit of physician work, allowing pricing variation to be assessed on a more comparable basis across procedures.

Medicaid-to-Medicare Index (MMI) was used as a measure of relative payment parity. An MMI value of 1.0 denotes equivalent reimbursement under Medicaid and Medicare for a given service, whereas values below and above 1.0 indicate that Medicaid reimbursement is lower or higher than Medicare reimbursement, respectively. Because Medicaid reimbursement policies are established independently by individual states, the MMI serves as a useful tool for evaluating geographic variation in payment rates and identifying disparities across state Medicaid programs. Given that Medicaid predominantly insures low-income individuals and persons with disabilities, differences in MMI values may also reflect broader structural inequities affecting access to healthcare among vulnerable populations. For the purposes of this analysis, MMI was calculated as the ratio of the Medicaid reimbursement amount to the corresponding Medicare reimbursement amount for each procedure.

In contrast to physician professional fees, which are reported by Medicare at the level of the MAC locality and can be readily identified for individual CPT codes, facility reimbursement data are not available in a comparable geographic format. Medicare does not provide state-specific payment schedules for hospital outpatient department (HOPD) or ambulatory surgical center (ASC) facility services on a CPT-code basis. Rather, facility payments are determined through institution-specific methodologies that incorporate numerous factors, including local wage adjustments, rural versus urban status, facility characteristics, and case-dependent payment modifications. Consequently, reimbursement for the same procedure may vary substantially between facilities, even within a single state. We considered estimating state-level HOPD and ASC reimbursement by applying geographic wage adjustments to nationally published facility payment rates. However, such an approach would largely reproduce differences attributable to the wage index itself rather than capture true variation in Medicare facility reimbursement. Because facility payments are influenced by multiple factors beyond regional labor costs, wage-adjusted estimates would not accurately reflect actual reimbursement at the state level and could lead to misleading comparisons. Accordingly, facility fee analyses were not included in the present study.

In addition to evaluating reimbursement rates, we examined longitudinal trends in Medicare utilization and spending for all four CPT codes (64555, 64575, 64590, and 64553). Data were obtained from the Medicare Physician & Other Practitioners by Geography and Service dataset, from which several utilization and payment metrics were extracted, including total services (the number of services reported on final-action claims), total rendering providers (the number of clinicians performing a given procedure), total beneficiaries (the number of unique Medicare beneficiaries receiving the service), and average standardized Medicare payment amount. The latter represents the average Medicare payment for a service after adjustment for beneficiary cost-sharing obligations, including deductibles and coinsurance, as well as CMS payment standardization procedures. This dataset contains annual service utilization and expenditure information from 2013 through 2024 for both facility and non-facility practice settings. Consistent with the Medicaid reimbursement data analyzed in the present study, Medicare utilization and payment data were extracted separately for facility and non-facility settings for CPT 64555, CPT 64590, and CPT 64553. Only facility-setting data were extracted for CPT 64575, which is performed exclusively in facility settings. These settings encompass locations such as hospital outpatient departments and ambulatory surgical centers, among others. A complete listing of Place of Service codes classified as “facility” within the dataset is provided in eTable 2.

### 2.3 Outcomes of interest

Primary study endpoints included Medicaid and Medicare professional fee reimbursement rates, Medicaid-to-Medicare reimbursement ratios (MMI), and reimbursement disparities expressed as dollars per wRVU for each selected procedure code across individual states. These measures were chosen to characterize both absolute and relative differences in physician payment between public insurance programs. Secondary analyses examined Medicare utilization and spending metrics, including service volume, number of rendering providers, beneficiary counts, and average standardized Medicare payment amounts.

### 2.4 Statistical analysis

Summary statistics for Medicaid and Medicare physician reimbursement included the median, minimum, maximum, 25th percentile, 75th percentile, and interquartile range (IQR). Medicaid reimbursement was evaluated both before and after adjustment using the Medicare Wage Index, and corresponding MMI values were calculated for each state under both approaches. Median, minimum, and maximum MMI values were subsequently reported. Differences in reimbursement between Medicaid and Medicare were assessed on a state-by-state basis by calculating the absolute payment differential for each CPT code and normalizing the result by the procedure's assigned wRVU value.

To evaluate longitudinal patterns in utilization and spending, Medicare service and payment data from 2013 to 2024 were extracted from the Medicare Physician & Other Practitioners by Geography and Service dataset. Temporal trends were visualized graphically, and percent changes over the study interval were calculated for all utilization and expenditure measures.

## 3 Results

### 3.1 Data availability and extraction

State-level data completeness for each outcome is summarized in Table 1. Complete Medicare professional fee and wage index data were obtained for all 50 states. Medicaid professional fee data, by contrast, could not be fully captured for every state due to variability in how individual state programs structure fee schedules, report reimbursement rates, and respond to verification requests. Medicaid physician professional fee data in facility settings were available for 37 states for CPT 64555, 46 for CPT 64575, 47 for CPT 64590, and 33 for CPT 64553. Non-facility data were available for 30 states for CPT 64555, 21 states for CPT 64590, and 27 states for CPT 64553. State-by-state baseline and wage-adjusted Medicaid rates for all four CPT codes are provided in eTable 3, while the corresponding Medicare professional fee rates by state appear in eTable 4.

**Table 1:** Data availability of selected CPT codes.

CPT Code	Medicare Reimbursement	Wage Index	Medicaid Reimbursement
**64555 (Facility)**	50 states	50 states	37 states *2 states: Fee schedule not compatible with study design.* *11 states: Facility rate not included on publicly available fee schedule.*
**64555 (Non-Facility)**	50 states	50 states	30 states *2 states: Fee schedule not compatible with study design.* *18 states: Non-facility rates not included on publicly available fee schedule.*
**64575 (Facility)**	50 states	50 states	46 states *2 states: Fee schedule not compatible with study design.* *2 states: Facility rate not included on publicly available fee schedule.*
**64590 (Facility)**	50 states	50 states	47 states *2 states: Fee schedule not compatible with study design.* *1 state: Facility rate not included on publicly available fee schedule.*
**64590 (Non-Facility)**	50 states	50 states	21 states *2 states: Fee schedule not compatible with study design.* *27 states: Non-facility rates not included on publicly available fee schedule.*
**64553 (Facility)**	50 states	50 states	33 states *2 states: Fee schedule not compatible with study design.* *15 states: Facility rate not included on publicly available fee schedule.*
**64553 (Non-Facility)**	50 states	50 states	27 states *2 states: Fee schedule not compatible with study design.* *21 states: Non-facility rates not included on publicly available fee schedule.*

Data availability for selected Current Procedural Terminology (CPT) codes. Due to state-to-state variability in fee schedule structure, reimbursement rate reporting, and responsiveness to verification inquiries, a number of states were excluded from our analysis of Medicaid reimbursement rates. 64555 = Percutaneous implantation of neurostimulator electrode array; peripheral nerve. 64575 = Open implantation of neurostimulator electrode array; peripheral nerve. 64590 = Insertion or replacement of peripheral neurostimulator pulse generator or receiver. 64553 = Percutaneous implantation of neurostimulator electrode array; cranial nerve.

### 3.2 National Medicaid and Medicare reimbursement

Median Medicaid physician professional fee reimbursement was lower than Medicare for all four CPT codes, in both facility and non-facility settings where applicable (Table 2). After wage adjustment, Medicaid reimbursed 32.1% below Medicare for CPT 64555 (facility), 27.3% below for CPT 64555 (non-facility), 14.0% below for CPT 64575 (facility), 19.2% below for CPT 64590 (facility), 27.3% below for CPT 64590 (non-facility), 26.8% below for CPT 64553 (facility), and 36.7% below for CPT 64553 (non-facility). The median dollar differential per wRVU was largest in magnitude for CPT 64553 (non-facility, -$193.36), followed by CPT 64555 (non-facility, -$81.96). The smallest differentials were observed for CPT 64575 (facility, -$7.58) and CPT 64590 (facility, -$7.69). State-level physician professional fee differentials are reported in eTable 5.

**Table 2:** Analysis of national medicaid, wage-adjusted medicaid, and medicare reimbursement for CPT codes 64555, 64575, 64590, and 64553 in 2026.

		Medicaid ($)	Wage-Adjusted Medicaid ($)	Medicare ($)	Dollar Difference per wRVU
**CPT 64555 (Facility)**	**Median *(*25th *percentile-*75th *percentile)***	202.47 (153.14-255.13)	195.87 (133.27-250.77)	288.67 (281.45-297.26)	−15.78 (−23.68 to −6.08)
	**Minimum**	42	47.47	272.29	−45.60
	**Maximum**	444.84	447.91	384.73	26.86
**CPT 64555 (Non-Facility)**	**Median *(*25th *percentile-*75th *percentile)***	1732.23 (1422.13-2009.40)	1584.52 (1401.61-2159.25)	2178.64 (2056.43-2285.72)	−81.96 (−131.61 to −35.82)
	**Minimum**	157.11	142.90	1929.79	−375.14
	**Maximum**	3099.68	3496.13	2590.92	144.68
**CPT 64575 (Facility)**	**Median *(*25th *percentile-*75th *percentile)***	257.23 (211.93-284.86)	246.49 (204.70-305.49)	286.65 (277.76-297.49)	−7.58 (−18.41-2.41)
	**Minimum**	84.00	94.95	264.57	−49.66
	**Maximum**	532.60	600.72	363.45	54.38
**CPT 64590 (Facility)**	**Median *(*25th *percentile-*75th *percentile)***	215.84 (165.70-288.45)	211.02 (162.65-286.96)	261.18 (252.53-268.47)	−7.69 (−20.79-6.49)
	**Minimum**	81.85	73.97	244.26	−39.86
	**Maximum**	688.57	776.64	343.90	72.21
**CPT 64590 (Non-Facility)**	**Median *(*25th *percentile-*75th *percentile)***	341.95 (257.96-356.50)	303.09 (278.89-397.59)	417.15 (404.21-436.31)	−18.82 (−35.10 to −7.82)
	**Minimum**	136.70	123.53	382.84	−54.53
	**Maximum**	563.72	567.61	515.71	27.28
**CPT 64553 (Facility)**	**Median *(*25th *percentile-*75th *percentile)***	305.41 (183.65-378.89)	319.70 (175.75-355.75)	437.04 (406.21-454.82)	−21.42 (−41.03 to −6.78)
	**Minimum**	27.55	41.07	385.65	−70.67
	**Maximum**	2819.69	2827.02	531.15	405.77
**CPT 64553 (Non-Facility)**	**Median *(*25th *percentile-*75th *percentile)***	3233.95 (2136.07-4268.61)	3004.94 (2423.50-4416.50)	4746.77 (4452.91-4974.01)	−193.36 (−517.04 to −48.79)
	**Minimum**	142.22	184.56	4158.02	−920.26
	**Maximum**	7241.04	8167.17	5645.39	341.35

Medicare Wage Index-Adjusted Medicaid reimbursement (Wage-Adjusted Medicaid) was calculated by multiplying Medicaid reimbursement by Medicare Wage Index for a given state. Dollar difference = median value of (Medicaid reimbursement - Medicare reimbursement) across all states. 64555 = Percutaneous implantation of neurostimulator electrode array; peripheral nerve. 64575 = Open implantation of neurostimulator electrode array; peripheral nerve. 64590 = Insertion or replacement of peripheral neurostimulator pulse generator or receiver. 64553 = Percutaneous implantation of neurostimulator electrode array; cranial nerve.

### 3.3 Interstate comparisons

Substantial interstate heterogeneity in physician professional fee reimbursement was observed across all four codes and in both settings (eTable 6). Wage-adjusted Medicaid reimbursement fell below Medicare in 86.5% of states for CPT 64555 (facility), 80.0% for CPT 64555 (non-facility), 67.4% for CPT 64575 (facility), 61.7% for CPT 64590 (facility), 81.0% for CPT 64590 (non-facility), 81.8% for CPT 64553 (facility), and 81.5% for CPT 64553 (non-facility) (Fig. 2, Fig. 3, eFig. 1, eFig. 2, eFig. 3, eFig. 4). Among states with data in facility settings across all four codes, only Arizona, Delaware, Hawaii, Montana, and South Dakota reimbursed at or above Medicare parity for every code. Within most states, wage-adjusted MMI values were closely aligned across procedures rather than varying independently by code. However, this pattern was not universal, and some states showed disproportionately low relative professional fee reimbursement for specific codes rather than uniformly low Medicaid rates overall.

**Fig. 2:**
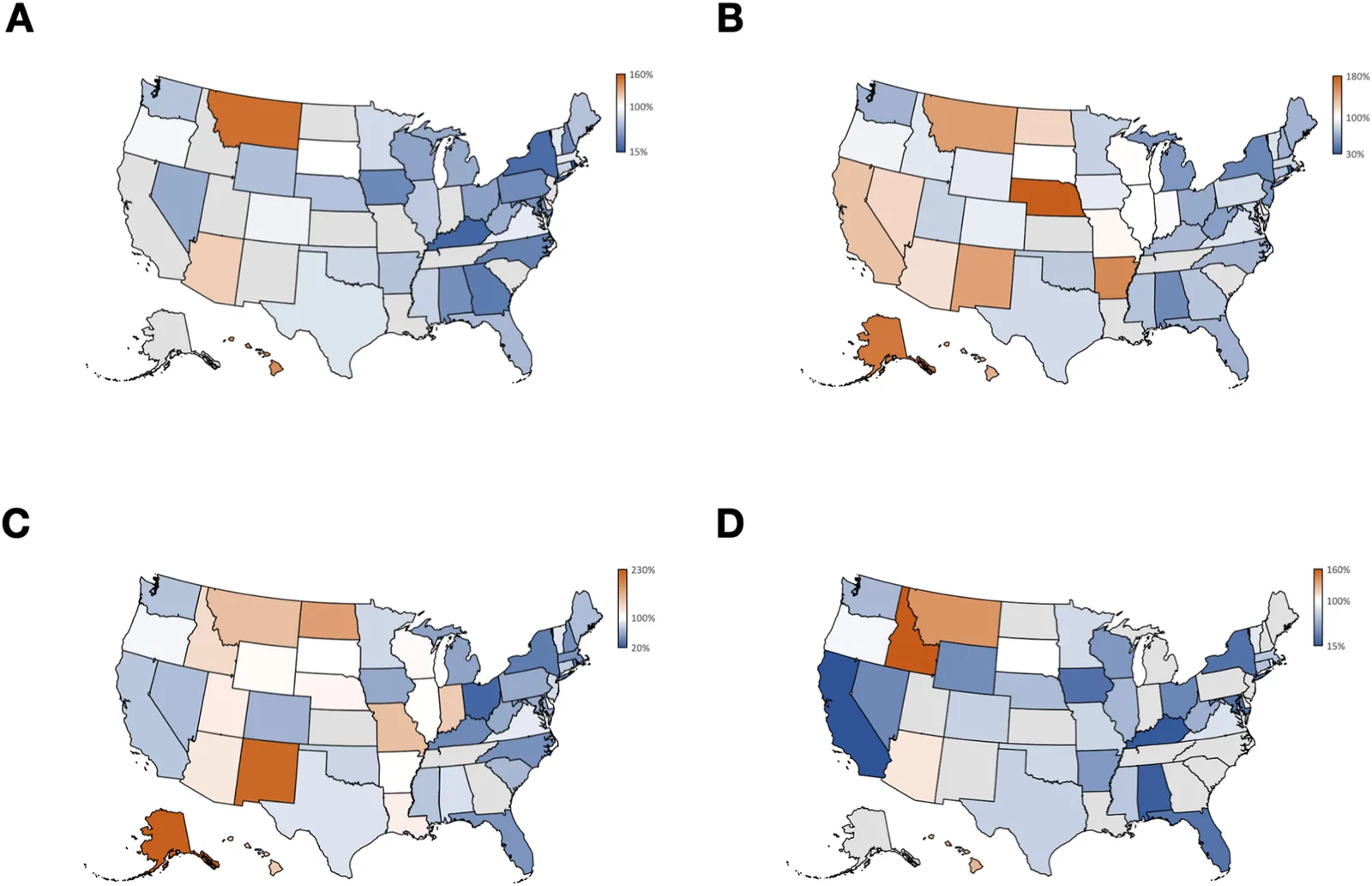
Medicaid to Medicare Reimbursement Ratio for CPT Codes 64555, 64575, 64590, and 64553 (Facility) A heatmap of the United States representing relative Medicaid reimbursement by state for CPT codes 64555 (A), 64575 (B), 64590 (C), and 64553 (D) in facility settings, as a percentage of Medicare reimbursement. Orange shades represent states where Medicaid reimbursement exceeded Medicare reimbursement (Medicaid-Medicare Fee Indices greater than 1), while blue shades represent states where Medicaid reimbursement was less than Medicare reimbursement (MMIs less than 1). States shaded gray were excluded from this analysis or lacked a facility setting reported rate for the given CPT code (see Methods).

**Fig. 3:**
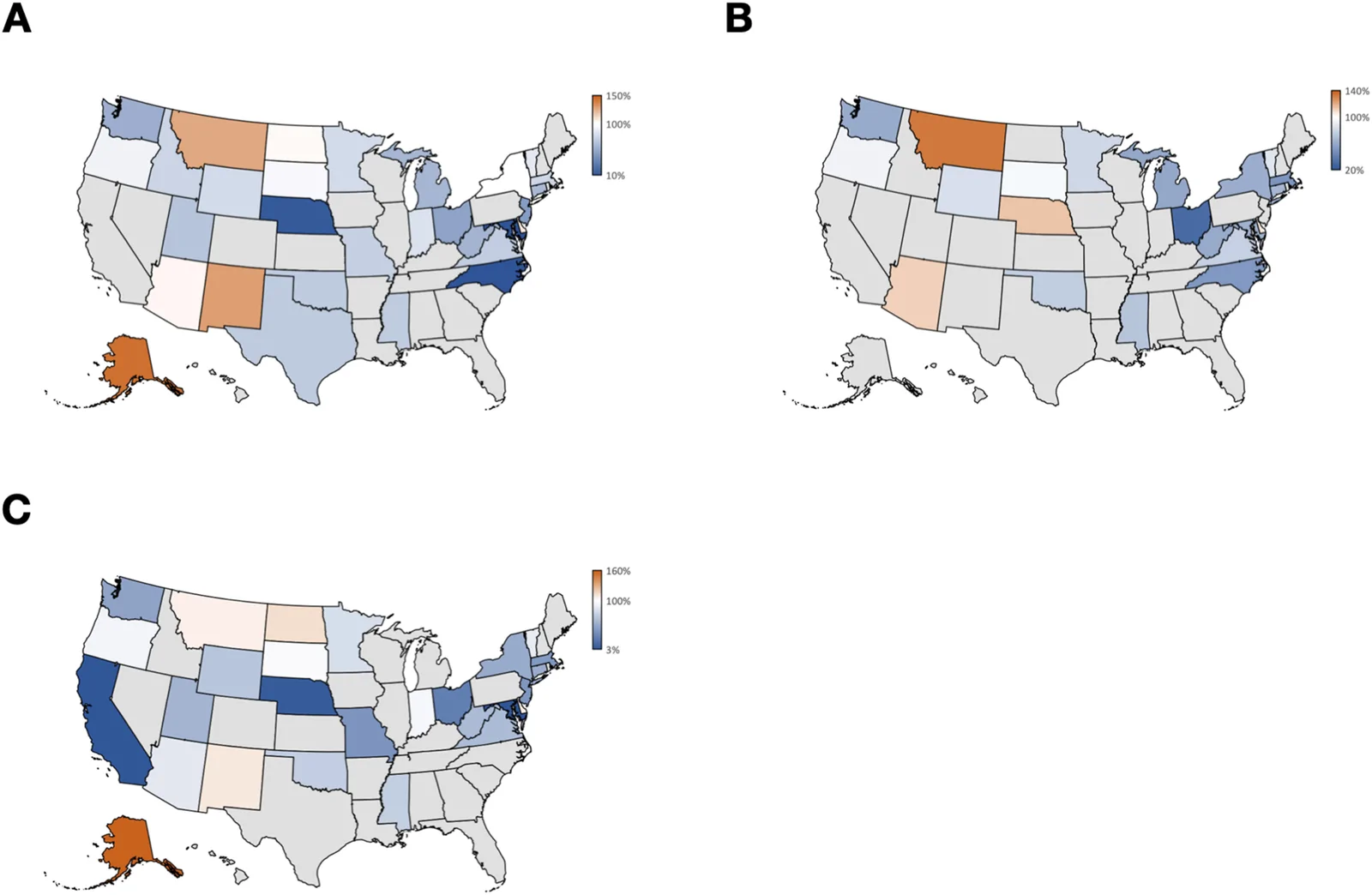
Medicaid to Medicare Reimbursement Ratio for CPT Codes 64555, 64590, and 64553 (Non-Facility) A heatmap of the United States representing relative Medicaid reimbursement by state for CPT codes 64555 (A), 64590 (B), and 64553 (C) in non-facility settings, as a percentage of Medicare reimbursement. CPT 64575 is not included because it is performed exclusively in facility settings. Orange shades represent states where Medicaid reimbursement exceeded Medicare reimbursement (Medicaid-Medicare Fee Indices greater than 1), while blue shades represent states where Medicaid reimbursement was less than Medicare reimbursement (MMIs less than 1). States shaded gray were excluded from this analysis or lacked a non-facility setting reported rate for the given CPT code (see Methods).

**Fig. 4:**
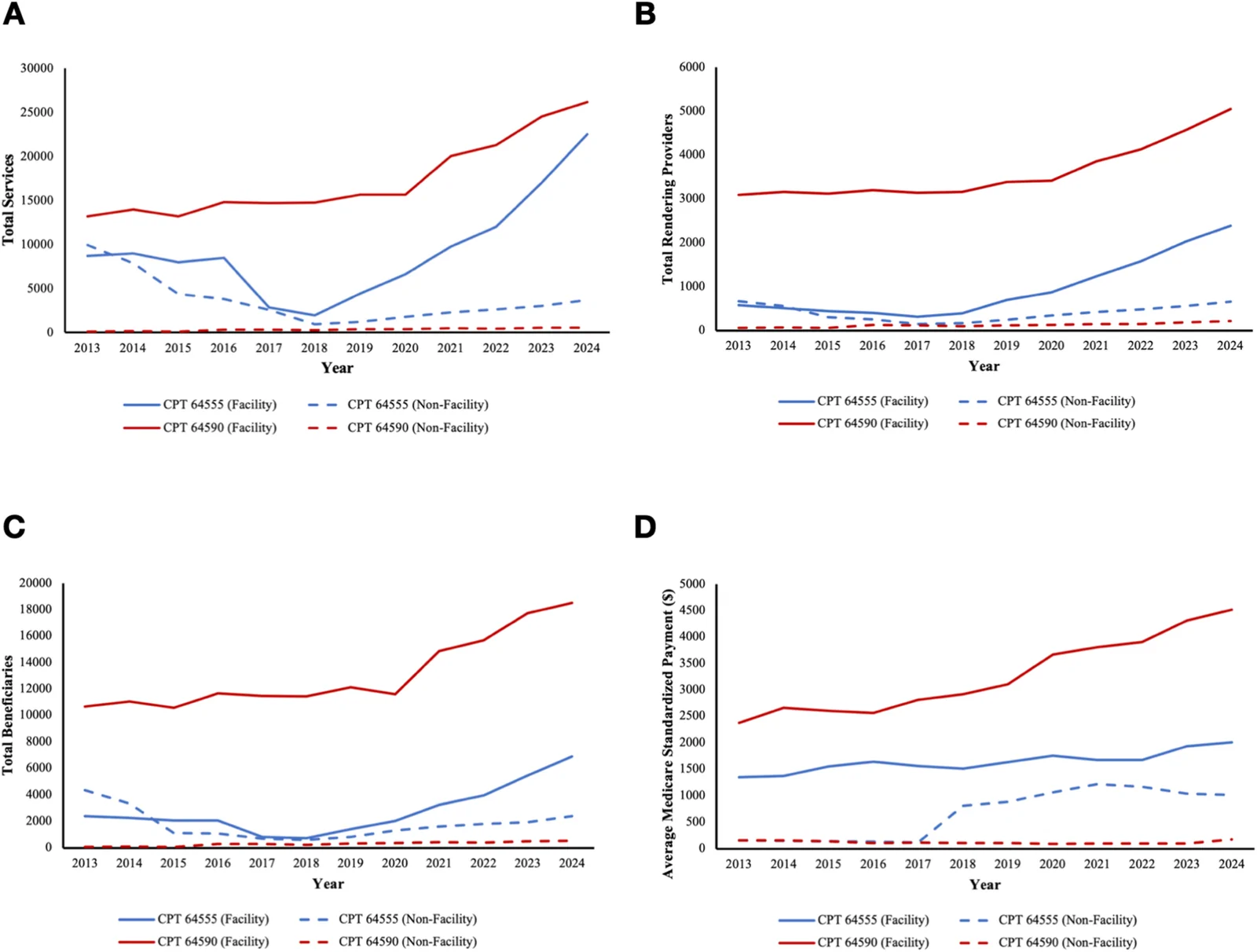
Trends in Medicare Service Utilization, Rendering Providers, Beneficiaries, and Average Standardized Payment for CPT Codes 64555 and 64590 Between 2013 and 2024 Trends in total services (A), total rendering providers (B), total beneficiaries (C), and average Medicare standardized payment (D) for CPT codes 64555 and 64590 between 2013 and 2024 in facility and non-facility settings. CPT 64575 and CPT 64553 are not included because non-facility utilization data for these two codes were reported inconsistently across the analyzed time period, including no non-facility claims reported for either code after 2021. “Total services” reflects the number of procedures billed under a given code in a given year; “total rendering providers” reflects the number of unique providers (NPIs) billing at least one service for that code; and “total beneficiaries” reflects the number of unique Medicare patients receiving the service, each in a given calendar year. “Average Medicare standardized payment,” as defined by CMS, is the average amount paid after beneficiary deductible and coinsurance amounts are deducted and after standardization removes geographic differences attributable to local wages and input prices, allowing observed differences to reflect variation in factors such as practice patterns and beneficiaries' access to care. 64555 = Percutaneous implantation of neurostimulator electrode array; peripheral nerve. 64590 = Insertion or replacement of peripheral neurostimulator pulse generator or receiver.

### 3.4 Medicare service utilization and expenditures

Medicare utilization and payment trends for 2013-2024 are reported for CPT 64555 and CPT 64590, in both facility and non-facility settings where applicable (Fig. 4). CPT 64575 and CPT 64553 were excluded from this analysis because their non-facility utilization data were sparse and discontinuous, with several years in which no claims were reported by CMS. Full utilization data are provided in eTable 7 – eTable 10. Total services in facility settings increased between 2013 and 2014 for both CPT 64555 (158.4%) and CPT 64590 (98.8%). Rendering providers and beneficiaries increased for both codes, more than tripling for CPT 64555 (308% and 189%, respectively) and increasing by roughly two-thirds for CPT 64590 (63% and 74%). Average standardized Medicare payment also increased for both codes, rising 48.6% for CPT 64555 and 90.4% for CPT 64590.

## 4 Discussion

### 4.1 Overall findings

The magnitude of the Medicaid–Medicare reimbursement gap differed meaningfully across the CPT codes evaluated. CPT 64555, which represents percutaneous lead placement, showed the largest facility-setting shortfall— 32.1% below Medicare after wage adjustment, with Medicaid reimbursement below parity in 86.5% of states. CPT 64553, representing percutaneous cranial nerve lead placement, showed a comparably large shortfall (26.8% below Medicare, 81.8% of states below parity). Reimbursement for both percutaneous codes was also more heterogeneous across states than for CPT 64575 or CPT 64590.

CPT 64590, representing pulse generator implantation, showed a comparably smaller facility setting gap (19.2%) and the highest rate of parity or better among the codes billed in facility settings (38.3% of states). This distinction has practical relevance because percutaneous lead placement is the technique underlying most contemporary PNS implantation [3], reflecting the field's continued shift toward less invasive, outpatient-based approaches. Perhaps most notably, the code with the largest reimbursement shortfall – CPT 64555 for percutaneous lead placement – is also the one that has become central to contemporary PNS practice.

### 4.2 Implications for policy

Consistent with prior reimbursement research in spine and orthopedic procedures [16], [18], [24], three of the five highest-reimbursing states in our analysis — Arizona, Hawaii, and Montana — are large, geographically expansive states with comparatively limited specialist density. In assessing the nationwide distribution of specialists during the opioid epidemic, each state was identified by Yoo et al. (2022) as one in which there is a mismatch between demand for and supply of pain medicine physicians [25]. This trend holds across specialties: Adornato et al. (2011) reported that these states had 3.32 (Arizona), 3.32 (Hawaii), and 3.18 (Montana) neurologists per 100,000 population, compared with states like New York (5.74 per 100,000) and Rhode Island (5.60 per 100,000), which were among the states with the lowest MMI values in our study [26].

Two explanations are typically offered for the association between higher Medicaid reimbursement and rural setting and/or low specialist density. First, because Medicaid rate-setting occurs at the state level, legislatures facing access concerns may deliberately adopt higher base rates to encourage physician participation [27]. Second, states with provider shortages may raise reimbursement to remain competitive in recruiting and retaining specialists. Prior work has found that adjusting for regional wage differences does little to explain interstate variation in reimbursement, suggesting these differences reflect state policy choices rather than underlying cost structures [28]. Our findings are broadly consistent with this conclusion: wage adjustment altered MMI values only modestly, and the states at both the high and low ends of the distribution remained largely unchanged before and after adjustment.

Low MMI values carry implications for access to PNS therapy. Prior studies have suggested that lower Medicaid reimbursement may be associated with reduced physician participation and diminished access to care for Medicaid beneficiaries [29]. Reduced reimbursement relative to Medicare may reduce physician willingness to accept Medicaid patients for a procedure as resource-intensive as PNS implantation, a pattern documented in other specialty care contexts [30], [31], [32]. This concern is heightened by evidence that Medicaid enrollees already report worse baseline health status than patients with other coverage [33], rely more heavily on emergency care and less on outpatient visits relative to Marketplace enrollees [34], and experience later diagnosis of serious conditions such as cancer [35]. Postoperative outcomes research similarly shows higher complication rates among Medicaid-insured surgical patients [36], [37]. The largest reimbursement shortfall in this analysis was concentrated in the most commonly performed PNS code, which may disproportionately affect overall adoption and economic viability of PNS programs under Medicaid.

Several policy approaches, informed by the patterns described here, could support the narrowing of reimbursement gaps. A federally imposed reimbursement floor could reduce the degree of variation in state Medicaid pricing by benchmarking against Medicare reimbursement rates. Section 1202 of the Affordable Care Act implemented such a mechanism for primary care services and saw increases in service availability [38], [39]. Moreover, routine and public reporting of MMI values by CPT code would improve transparency and make it easier to track whether such interventions prove effective.

### 4.3 Implications for clinical practice and research

Utilization trends provide additional context for these reimbursement patterns. Between 2013 and 2024, rendering providers for CPT 64555 grew considerably faster than beneficiaries (308% versus 189%), likely reflecting myriad factors including advancements in technology, increasing evidence base, and training of physicians [40]. This is a different relationship than the one observed for CPT 64590, where provider and beneficiary growth were more proportionate (63% versus 74%). Average standardized payment also increased for both codes despite these divergent utilization trends, rising 90.4% for CPT 64590 and 48.6% for 64555. Together, these trends indicate that CPT 64555 and CPT 64590 have followed distinct utilization and payment trajectories over the past decade, despite both falling under the broader category of PNS reimbursement.

For researchers, these findings support evaluating PNS reimbursement at the level of individual procedure codes rather than as a single aggregate measure. Future work should examine how these code-specific gaps evolve as percutaneous techniques continue to standardize, and whether reimbursement differentials measurably influence provider participation or procedural volume at the state level. Given the limited availability and inconsistency of historical Medicaid fee schedule data, improved standardization of public reporting would substantially strengthen the feasibility of such longitudinal analyses.

### 4.4 Limitations

This study has several limitations. State-level Medicaid reimbursement data are incomplete due to gaps in availability, inability to verify outlier values, and lack of comparability among state models. Use of locality-averaged Medicare rates for multi-locality states may not fully account for differences in population across localities, a limitation also noted in prior Medicaid reimbursement analyses [16], [17], [18]. Published Medicaid rates may not reflect actual reimbursement received, given the potential influence of managed care contracts, supplemental payments, and other adjustments not captured in publicly available fee schedules. This analysis is also limited to physician professional fees and does not capture facility payments, which for implant-based therapies such as PNS often represent a substantial share of total procedural cost. Non-facility Medicare utilization data for CPT 64575 and CPT 64553 were reported for only a subset of years between 2013 and 2024, with several years missing entirely and no non-facility claims reported for either code after 2021. Given this gap in data availability, these two codes were excluded from analysis of utilization trends to avoid presenting conclusions derived from small claims volumes.

Finally, because this analysis reflects reimbursement at a single point in time, it cannot characterize how the Medicaid-Medicare gap for PNS procedures is trending over time.

## 5 Conclusion

Medicaid reimbursement for common PNS procedural codes was consistently lower than Medicare, with the gap and degree of interstate variability differing by procedure and site of service. The shortfall was largest for the two percutaneous codes, CPT 64555 (32.1% below Medicare in facility settings, 27.3% in non-facility settings) and CPT 64553 (26.8% facility, 36.7% non-facility), compared with 14.0% for CPT 64575 and 19.2% for CPT 64590. CPT 64553, added to this analysis as a post-hoc extension, showed a comparably heterogeneous reimbursement gap. Because percutaneous approaches now predominate in PNS practice, this disparity may exert outsized influence on procedural economics and provider incentives under Medicaid. Medicare utilization trends from 2013 to 2024 showed substantial growth in services, providers, and beneficiaries for CPT 64555 and CPT 64590, the two most frequently billed codes in this analysis, in addition to rising payment. In particular, provider growth for percutaneous lead placement substantially outpaced beneficiary growth (308% vs. 189%), likely reflecting myriad factors including technological advancements and an expanding evidence base. If similar trends hold within Medicaid networks, reimbursement levels – rather than provider availability – may increasingly limit access for beneficiaries in lower-paying states. While not addressed in this study, facility payments from public payers may further compound this issue if insufficient to cover equipment costs. As PNS utilization continues to rise, procedure-specific benchmarking, targeted payment adjustments, and greater transparency in Medicaid fee schedules could help reduce disparities in access to neuromodulation therapies for vulnerable populations.

## Permission to reproduce material from other sources

Not applicable.

## Clinical trial registration

Not applicable.

## Patient consent

Not applicable.

## Author contributions

EJM was involved in study conception and design, data extraction, data analysis, and manuscript composition. JK was involved in study conception and design and manuscript editing. DAP was involved in study design and manuscript editing. RSD was involved in study conception and design, manuscript composition, and supervision of all aspects of the study.

## Ethics approval statement

As the current study uses data from publicly available databases, ethical approval was deemed as exempt.

## Funding

The authors have not declared a specific grant for this research from any funding agency in the public, commercial, or not-for-profit sectors.

## Conflicts of interest

RSD has received investigator-initiated grant funding paid to his institution from Nevro Corp and Saol Therapeutics. DAP has consulted for Avanos, Boston Scientific, Medtronic, and SI Bone, and holds a patent with SI Bone. Pain Diagnostics and Interventional Care has received research support from Avanos, Boston Scientific, Brixton, Medtronic, and Saol Therapeutics. Other authors declare no conflict of interest.
