# Supplementary material for: National and state-level analysis of peripheral nerve stimulation reimbursement patterns
Source: Interv Pain Med. 2026 Oct 21;5(4):100826. doi: 10.1016/j.inpm.2026.100826 (PMC13718633; doi:10.1016/j.inpm.2026.100826)
Supplement: Supplementary file 1 [file mmc1.docx]

**Appendix A: Supplemental Content**

**National and State-level Analysis of Peripheral Nerve Stimulation Reimbursement Patterns**

Evan J. Mizerak, ScB^1^, Jay Karri, MD, MPH^2^, David A. Provenzano, MD^3^, Ryan S. D’Souza, MD^5^

**eTable 1 –** Included Codes with Corresponding wRVUs

**eTable 2 –** Place of Service Codes Captured by Facility and Non-Facility Designations in Medicare - Physician & Other Practitioners by Geography and Service Dataset

**eTable 3 –** Analysis of Medicaid Reimbursement for CPT Codes 64555, 64575, 64590, and 64553 in 2026

**eTable 4 –** Analysis of Medicare Reimbursement for CPT Codes 64555, 64575, 64590, and 64553 in 2026

**eTable 5 –** Dollar Difference per wRVU between Medicaid and Medicare Reimbursement for CPT Codes 64555, 64575, 64590, and 64553

**eTable 6 –** Analysis of relative Medicaid reimbursement for CPT Codes 64555, 64575, 64590, and 64553 by state as a fraction of corresponding Medicare rates

**eTable 7 –** Medicare Service Utilization, Rendering Providers, Beneficiaries, and Average Standardized Payment for CPT Code 64555 Between 2013 and 2024

**eTable 8 –** Medicare Service Utilization, Rendering Providers, Beneficiaries, and Average Standardized Payment for CPT Code 64590 Between 2013 and 2024

**eTable 9 –** Medicare Service Utilization, Rendering Providers, Beneficiaries, and Average Standardized Payment for CPT Code 64575 Between 2013 and 2024

**eTable 10 –** Medicare Service Utilization, Rendering Providers, Beneficiaries, and Average Standardized Payment for CPT Code 64553 Between 2013 and 2024

**eFigure 1 –** Wage-Adjusted Medicaid Reimbursement Rates for CPT Codes 64555, 64575, 64590, and 64553 (Facility)

**eFigure 2 –** Wage-Adjusted Medicaid Reimbursement Rates for CPT Codes 64555, 64590, and 64553 (Non-Facility)

**eFigure 3 –** Medicare Reimbursement Rates for CPT Codes 64555, 64575, 64590, and 64553 (Facility)

**eFigure 4 –** Medicare Reimbursement Rates for CPT Codes 64555, 64590, and 64553 (Non-Facility)

**eTable 1: Included Codes with Corresponding wRVUs**

| **CPT Code** | **Description** | **wRVUs** |
| --- | --- | --- |
| 64555 | Percutaneous implantation of neurostimulator electrode array; peripheral nerve | 5.62 |
| 64575 | Open implantation of neurostimulator electrode array; peripheral nerve | 4.31 |
| 64590 | Insertion or replacement of peripheral neurostimulator pulse generator or receiver | 4.97 |
| 64553 | Percutaneous implantation of neurostimulator electrode array; cranial nerve | 5.98 |

CPT = Current Procedural Terminology. wRVUs = work Relative Value Units. Data are for 2026.

**eTable 2: Place of Service Codes Captured by Facility and Non-Facility Designations in Medicare - Physician & Other Practitioners by Geography and Service Dataset**

| **Place of Service Code** | **Place of Service Description** | **Place of Service** |
| --- | --- | --- |
| 01 | Pharmacy | Non-Facility |
| 02 | Telehealth Provided Other than in Patient’s Home | Non-Facility |
| 03 | School | Non-Facility |
| 04 | Homeless Shelter | Non-Facility |
| 05 | Indian Health Service Free-standing Facility | Non-Facility |
| 06 | Indian Health Service Provider-based Facility | Non-Facility |
| 07 | Tribal 638 Free-standing Facility | Non-Facility |
| 08 | Tribal 638 Provider-based Facility | Non-Facility |
| 09 | Prison/ Correctional Facility | Non-Facility |
| 10 | Telehealth Provided in Patient’s Home | Non-Facility |
| 11 | Office | Non-Facility |
| 12 | Home | Non-Facility |
| 13 | Assisted Living Facility | Non-Facility |
| 14 | Group Home | Non-Facility |
| 15 | Mobile Unit | Non-Facility |
| 16 | Temporary Lodging | Non-Facility |
| 17 | Walk-in Retail Health Clinic | Non-Facility |
| 18 | Place of Employment-Worksite | Non-Facility |
| 19 | Off Campus-Outpatient Hospital | Non-Facility |
| 20 | Urgent Care Facility | Non-Facility |
| 25 | Birthing Center | Non-Facility |
| 27 | Outreach Site/ Street | Non-Facility |
| 32 | Nursing Facility | Non-Facility |
| 33 | Custodial Care Facility | Non-Facility |
| 49 | Independent Clinic | Non-Facility |
| 50 | Federally Qualified Health Center | Non-Facility |
| 54 | Intermediate Care Facility/Mentally Retarded | Non-Facility |
| 55 | Residential Substance Abuse Treatment Facility | Non-Facility |
| 57 | Non-residential Substance Abuse Treatment Facility | Non-Facility |
| 58 | Non-residential Opioid Treatment Facility | Non-Facility |
| 60 | Mass Immunization Center | Non-Facility |
| 62 | Comprehensive Outpatient Rehabilitation Facility | Non-Facility |
| 65 | End-Stage Renal Disease Treatment Facility | Non-Facility |
| 71 | Public Health Clinic | Non-Facility |
| 72 | Rural Health Clinic | Non-Facility |
| 81 | Independent Laboratory | Non-Facility |
| 99 | Other Place of Service | Non-Facility |
| 21 | Inpatient Hospital | Facility |
| 22 | Outpatient Hospital | Facility |
| 23 | Emergency Room – Hospital | Facility |
| 24 | Ambulatory Surgical Center | Facility |
| 26 | Military Treatment Facility | Facility |
| 31 | Skilled Nursing Facility | Facility |
| 34 | Hospice | Facility |
| 41 | Ambulance - Land | Facility |
| 42 | Ambulance – Air or Water | Facility |
| 51 | Inpatient Psychiatric Facility | Facility |
| 52 | Psychiatric Facility-Partial Hospitalization | Facility |
| 53 | Community Mental Health Center | Facility |
| 56 | Psychiatric Residential Treatment Center | Facility |
| 61 | Comprehensive Inpatient Rehabilitation Facility | Facility |

Place of service codes captured by “facility” and “non-facility” designations in the Medicare Physician & Other Practitioners by Geography and Service dataset. For national-level data on Medicare service utilization, beneficiaries, rendering providers, and average standardized payment, only “Facility” settings were considered.

**eTable 3: Analysis of Medicaid Reimbursement for CPT Codes 64555, 64575, 64590, and 64553 in 2026**

| **State** | **CPT 64555 (Facility)** | | **CPT 64555 (Non-Facility)** | | **CPT 64575 (Facility)** | | **CPT 64590 (Facility)** | | **CPT 64590 (Non-Facility)** | | **CPT 64553 (Facility)** | | **CPT 64553 (Non-Facility)** | |
| --- | --- | --- | --- | --- | --- | --- | --- | --- | --- | --- | --- | --- | --- | --- |
|  | **Baseline** | **Wage-Adjusted** | **Baseline** | **Wage-Adjusted** | **Baseline** | **Wage-Adjusted** | **Baseline** | **Wage-Adjusted** | **Baseline** | **Wage-Adjusted** | **Baseline** | **Wage-Adjusted** | **Baseline** | **Wage-Adjusted** |
| **Alabama** | 154.00 | 121.89 | N/A | N/A | 174.00 | 137.72 | 263.00 | 208.16 | N/A | N/A | 98.00 | 77.57 | N/A | N/A |
| **Alaska** | N/A | N/A | 3,099.68 | 3,496.13 | 532.60 | 600.72 | 688.57 | 776.64 | N/A | N/A | N/A | N/A | 7,241.04 | 8,167.17 |
| **Arizona** | 340.09 | 340.51 | 2,222.23 | 2,224.97 | 331.15 | 331.56 | 309.34 | 309.72 | 464.19 | 464.76 | 473.48 | 474.06 | 4,040.71 | 4,045.69 |
| **Arkansas** | 218.55 | 187.56 | N/A | N/A | 485.47 | 416.63 | 287.32 | 246.58 | N/A | N/A | 217.97 | 187.06 | N/A | N/A |
| **California** | N/A | N/A | N/A | N/A | 272.90 | 406.85 | 135.14 | 201.47 | N/A | N/A | 27.55 | 41.07 | 142.22 | 212.03 |
| **Colorado** | 263.31 | 275.84 | N/A | N/A | 261.12 | 273.55 | 163.70 | 171.49 | N/A | N/A | 324.71 | 340.17 | N/A | N/A |
| **Connecticut** | 201.42 | 232.80 | 1,295.06 | 1,496.81 | 197.60 | 228.38 | 182.58 | 211.02 | 257.96 | 298.15 | 289.43 | 334.52 | 2,363.21 | 2,731.37 |
| **Delaware** | 286.03 | 310.39 | 2,155.93 | 2,339.56 | 284.98 | 309.25 | 259.03 | 281.09 | 415.24 | 450.61 | 432.58 | 469.43 | 4,686.92 | 5,086.13 |
| **Florida** | 215.48 | 201.71 | N/A | N/A | 228.34 | 213.75 | 153.23 | 143.44 | N/A | N/A | 169.87 | 159.01 | N/A | N/A |
| **Georgia** | 117.79 | 99.56 | N/A | N/A | 261.42 | 220.96 | N/A | N/A | N/A | N/A | N/A | N/A | N/A | N/A |
| **Hawaii** | 329.53 | 422.42 | N/A | N/A | 325.79 | 417.63 | 298.14 | 382.19 | N/A | N/A | 435.24 | 557.93 | N/A | N/A |
| **Idaho** | N/A | N/A | 1,566.57 | 1,570.64 | 243.58 | 244.21 | 319.67 | 320.50 | N/A | N/A | 2,819.69 | 2,827.02 | N/A | N/A |
| **Illinois** | 220.63 | 223.10 | N/A | N/A | 307.51 | 310.96 | 289.59 | 292.84 | N/A | N/A | 327.00 | 330.66 | N/A | N/A |
| **Indiana** | N/A | N/A | 1,838.58 | 1,735.41 | 284.49 | 268.53 | 373.54 | 352.58 | N/A | N/A | N/A | N/A | 4,465.50 | 4,214.91 |
| **Iowa** | 125.71 | 112.18 | N/A | N/A | 274.51 | 244.96 | 162.25 | 144.78 | N/A | N/A | 126.35 | 112.75 | N/A | N/A |
| **Kansas** | Excluded | -- | Excluded | -- | Excluded | -- | Excluded | -- | Excluded | -- | Excluded | -- | Excluded | -- |
| **Kentucky** | 73.21 | 65.98 | N/A | N/A | 217.36 | 195.89 | 126.81 | 114.28 | N/A | N/A | 82.43 | 74.29 | N/A | N/A |
| **Louisiana** | N/A | N/A | N/A | N/A | N/A | N/A | 350.05 | 294.15 | N/A | N/A | N/A | N/A | N/A | N/A |
| **Maine** | 202.47 | 193.65 | N/A | N/A | 198.93 | 190.27 | 182.11 | 174.18 | N/A | N/A | N/A | N/A | N/A | N/A |
| **Maryland** | 139.72 | 133.27 | 204.87 | 195.41 | 268.35 | 255.96 | 173.18 | 165.18 | 292.53 | 279.02 | 151.51 | 144.51 | 193.50 | 184.56 |
| **Massachusetts** | 240.88 | 270.25 | 1,718.82 | 1,928.40 | 230.15 | 258.21 | 120.28 | 134.95 | 202.78 | 227.51 | 293.31 | 329.07 | 2,014.98 | 2,260.67 |
| **Michigan** | 187.44 | 170.54 | 1,417.94 | 1,290.06 | 187.23 | 170.34 | 170.19 | 154.84 | 273.07 | 248.44 | N/A | N/A | N/A | N/A |
| **Minnesota** | 217.67 | 227.85 | 1,747.32 | 1,829.08 | 216.38 | 226.50 | 194.00 | 203.08 | 321.88 | 336.94 | 305.41 | 319.70 | 3,785.65 | 3,962.78 |
| **Mississippi** | 266.60 | 212.50 | 1,745.64 | 1,391.40 | 253.54 | 202.09 | 224.89 | 179.25 | 349.90 | 278.89 | 365.16 | 291.06 | 3,769.98 | 3,004.94 |
| **Missouri** | N/A | N/A | 1,704.69 | 1,530.16 | 327.75 | 294.19 | 439.30 | 394.32 | N/A | N/A | 382.95 | 343.74 | 1,944.68 | 1,745.58 |
| **Montana** | 444.84 | 447.91 | 2,790.26 | 2,809.51 | 431.99 | 434.97 | 402.06 | 404.83 | 563.72 | 567.61 | 623.71 | 628.01 | 5,071.16 | 5,106.15 |
| **Nebraska** | 197.92 | 189.07 | 266.76 | 254.83 | 503.88 | 481.35 | 279.32 | 266.83 | 474.24 | 453.03 | 266.89 | 254.96 | 340.86 | 325.62 |
| **Nevada** | 157.71 | 169.14 | N/A | N/A | 323.62 | 347.08 | 167.70 | 179.86 | N/A | N/A | 163.87 | 175.75 | N/A | N/A |
| **New Hampshire** | 113.68 | 124.22 | N/A | N/A | 183.46 | 200.47 | 113.70 | 124.24 | N/A | N/A | N/A | N/A | N/A | N/A |
| **New Jersey** | N/A | N/A | 1,038.39 | 1,190.13 | 157.17 | 180.14 | 205.81 | 235.89 | N/A | N/A | N/A | N/A | 1,891.20 | 2,167.57 |
| **New Mexico** | N/A | N/A | 2,873.15 | 2,668.29 | 452.61 | 420.34 | 624.32 | 579.81 | N/A | N/A | N/A | N/A | 5,236.32 | 4,862.97 |
| **New York** | 70.75 | 84.80 | 2,053.22 | 2,460.82 | 135.71 | 162.65 | 94.23 | 112.94 | 241.48 | 289.42 | 126.25 | 151.31 | 2,343.03 | 2,808.16 |
| **North Carolina** | 115.70 | 105.23 | 157.11 | 142.90 | 210.44 | 191.40 | 133.63 | 121.54 | 229.03 | 208.31 | N/A | N/A | N/A | N/A |
| **North Dakota** | N/A | N/A | 2,253.39 | 2,253.39 | 337.49 | 337.49 | 445.45 | 445.45 | N/A | N/A | N/A | N/A | 5,386.43 | 5,386.43 |
| **Ohio** | 166.29 | 150.27 | 1,162.94 | 1,050.93 | 204.04 | 184.39 | 81.85 | 73.97 | 136.70 | 123.53 | 192.93 | 174.35 | 1,326.80 | 1,199.01 |
| **Oklahoma** | 255.13 | 221.67 | 1,658.91 | 1,441.37 | 246.15 | 213.87 | 231.46 | 201.11 | 348.83 | 303.09 | 378.89 | 329.20 | 3,573.05 | 3,104.49 |
| **Oregon** | 233.30 | 279.40 | 1,810.21 | 2,167.91 | 232.87 | 278.89 | 210.22 | 251.76 | 341.95 | 409.52 | 345.58 | 413.87 | 3,933.30 | 4,710.52 |
| **Pennsylvania** | 125.92 | 124.14 | N/A | N/A | 250.93 | 247.38 | 162.41 | 160.11 | N/A | N/A | N/A | N/A | N/A | N/A |
| **Rhode Island** | 42.00 | 47.48 | N/A | N/A | 84.00 | 94.95 | 84.00 | 94.95 | N/A | N/A | N/A | N/A | N/A | N/A |
| **South Carolina** | N/A | N/A | N/A | N/A | N/A | N/A | 196.13 | 169.98 | N/A | N/A | N/A | N/A | N/A | N/A |
| **South Dakota** | 281.06 | 281.06 | 2,133.26 | 2,133.26 | 278.29 | 278.29 | 250.68 | 250.68 | 397.59 | 397.59 | 395.02 | 395.02 | 4,618.08 | 4,618.08 |
| **Tennessee** | Excluded | -- | Excluded | -- | Excluded | -- | Excluded | -- | Excluded | -- | Excluded | -- | Excluded | -- |
| **Texas** | 271.69 | 250.77 | 1,701.66 | 1,570.61 | 266.08 | 245.59 | 247.83 | 228.74 | N/A | N/A | 367.28 | 338.99 | N/A | N/A |
| **Utah** | N/A | N/A | 1,495.48 | 1,440.05 | 237.96 | 229.14 | 309.24 | 297.78 | N/A | N/A | N/A | N/A | 2,711.04 | 2,610.55 |
| **Vermont** | 240.81 | 246.88 | 1,877.93 | 1,925.25 | 239.37 | 245.40 | 215.84 | 221.28 | 352.60 | 361.49 | 347.01 | 355.75 | 4,071.72 | 4,174.33 |
| **Virginia** | 278.96 | 255.13 | 1,747.66 | 1,598.40 | 270.99 | 247.85 | 252.23 | 230.69 | 353.46 | 323.27 | 392.13 | 358.64 | 3,177.13 | 2,905.78 |
| **Washington** | 188.78 | 217.69 | 1,242.03 | 1,432.25 | 184.30 | 212.53 | 170.28 | 196.36 | 242.80 | 279.98 | 259.81 | 299.60 | 2,257.15 | 2,602.83 |
| **West Virginia** | 209.84 | 173.70 | 1,434.72 | 1,187.63 | 207.40 | 171.68 | 192.76 | 159.56 | 295.24 | 244.39 | 337.21 | 279.14 | 3,124.42 | 2,586.33 |
| **Wisconsin** | 153.14 | 148.28 | N/A | N/A | 284.41 | 275.38 | 267.99 | 259.49 | N/A | N/A | 191.43 | 185.35 | N/A | N/A |
| **Wyoming** | 195.87 | 195.87 | 1,784.62 | 1,784.62 | 260.92 | 260.92 | 268.47 | 268.47 | 356.50 | 356.50 | 183.65 | 183.65 | 3,233.95 | 3,233.95 |

64555 = Percutaneous implantation of neurostimulator electrode array; peripheral nerve. 64575 = Open implantation of neurostimulator electrode array; peripheral nerve. 64590 = Insertion or replacement of peripheral neurostimulator pulse generator or receiver. 64553 = Percutaneous implantation of neurostimulator electrode array; cranial nerve. Tennessee is not included in this analysis because there is no single fee schedule for the entire state. Instead, TennCare services are offered through managed care organizations (MCOs) in each region of the state, and each participating MCO maintains its own fee schedule.

**eTable 4: Analysis of Medicare Reimbursement for CPT Codes 64555, 64575, 64590, and 64553 in 2026**

| **State** | **CPT 64555 (Facility)** | **CPT 64555 (Non-Facility)** | **CPT 64575 (Facility)** | **CPT 64590 (Facility)** | **CPT 64590 (Non-Facility)** | **CPT 64553 (Facility)** | **CPT 64553 (Non-Facility)** |
| --- | --- | --- | --- | --- | --- | --- | --- |
| **Alabama** | 274.66 | 1,963.04 | 267.77 | 246.73 | 387.89 | 393.51 | 4,235.24 |
| **Alaska** | 384.73 | 2,439.72 | 363.45 | 343.90 | 515.71 | 523.84 | 5,199.78 |
| **Arizona** | 288.45 | 2,158.20 | 286.35 | 260.99 | 417.32 | 433.61 | 4,688.06 |
| **Arkansas** | 272.29 | 1,929.79 | 264.57 | 244.26 | 382.84 | 386.54 | 4,158.02 |
| **California** | 307.71 | 2,590.92 | 311.40 | 275.68 | 466.57 | 450.16 | 5,645.39 |
| **Colorado** | 297.50 | 2,350.56 | 298.38 | 268.42 | 440.07 | 445.56 | 5,117.11 |
| **Connecticut** | 308.44 | 2,386.58 | 311.06 | 281.20 | 454.95 | 485.44 | 5,214.06 |
| **Delaware** | 291.87 | 2,198.28 | 290.47 | 264.32 | 423.71 | 441.41 | 4,779.27 |
| **Florida** | 312.56 | 2,248.56 | 315.83 | 290.21 | 452.07 | 531.15 | 4,936.34 |
| **Georgia** | 294.02 | 2,134.83 | 292.51 | 268.39 | 422.30 | 460.07 | 4,648.65 |
| **Hawaii** | 297.67 | 2,491.59 | 301.23 | 267.09 | 450.52 | 438.36 | 5,430.41 |
| **Idaho** | 276.77 | 2,051.98 | 271.32 | 247.90 | 396.32 | 393.19 | 4,432.50 |
| **Illinois** | 309.30 | 2,173.75 | 310.82 | 287.09 | 442.97 | 522.65 | 4,765.02 |
| **Indiana** | 277.63 | 2,066.34 | 272.51 | 248.76 | 398.31 | 395.46 | 4,465.50 |
| **Iowa** | 274.86 | 2,040.42 | 268.94 | 245.67 | 393.28 | 385.96 | 4,403.31 |
| **Kansas** | 275.98 | 2,020.31 | 270.01 | 247.44 | 393.28 | 393.13 | 4,362.19 |
| **Kentucky** | 282.64 | 1,998.03 | 277.58 | 256.31 | 399.73 | 425.22 | 4,328.42 |
| **Louisiana** | 287.28 | 2,048.98 | 283.66 | 261.37 | 408.65 | 440.34 | 4,448.91 |
| **Maine** | 282.83 | 2,126.53 | 279.36 | 254.37 | 408.51 | 412.05 | 4,607.21 |
| **Maryland** | 301.56 | 2,313.14 | 302.63 | 274.17 | 442.35 | 467.13 | 5,044.28 |
| **Massachusetts** | 306.97 | 2,474.85 | 309.73 | 277.24 | 458.49 | 464.06 | 5,396.84 |
| **Michigan** | 296.52 | 2,108.39 | 295.26 | 272.14 | 423.63 | 475.02 | 4,597.75 |
| **Minnesota** | 282.81 | 2,268.34 | 281.04 | 251.93 | 417.93 | 396.50 | 4,914.37 |
| **Mississippi** | 276.80 | 1,938.16 | 269.98 | 249.88 | 388.78 | 405.73 | 4,185.99 |
| **Missouri** | 286.47 | 2,057.18 | 282.81 | 260.19 | 408.24 | 435.84 | 4,464.90 |
| **Montana** | 293.89 | 2,223.46 | 293.55 | 266.82 | 428.15 | 450.74 | 4,841.29 |
| **Nebraska** | 275.19 | 2,056.18 | 269.51 | 245.82 | 394.72 | 385.70 | 4,438.17 |
| **Nevada** | 290.78 | 2,222.28 | 289.87 | 262.88 | 424.37 | 437.02 | 4,831.96 |
| **New Hampshire** | 295.07 | 2,303.75 | 295.90 | 266.96 | 434.90 | 447.24 | 5,017.80 |
| **New Jersey** | 316.14 | 2,488.84 | 318.86 | 287.08 | 468.73 | 489.19 | 5,432.94 |
| **New Mexico** | 290.61 | 2,060.03 | 287.72 | 265.43 | 413.36 | 453.97 | 4,480.10 |
| **New York** | 319.83 | 2,472.46 | 323.34 | 292.35 | 472.33 | 509.95 | 5,408.05 |
| **North Carolina** | 281.12 | 2,081.40 | 276.79 | 252.95 | 403.46 | 409.34 | 4,505.72 |
| **North Dakota** | 282.42 | 2,211.99 | 279.90 | 252.39 | 413.72 | 400.92 | 4,791.46 |
| **Ohio** | 286.53 | 2,048.23 | 282.77 | 260.41 | 407.70 | 437.06 | 4,445.63 |
| **Oklahoma** | 280.32 | 2,003.42 | 274.91 | 253.25 | 397.32 | 414.27 | 4,335.03 |
| **Oregon** | 294.72 | 2,327.52 | 295.14 | 265.55 | 435.51 | 438.18 | 5,063.61 |
| **Pennsylvania** | 295.18 | 2,185.19 | 293.88 | 268.48 | 426.50 | 455.10 | 4,755.64 |
| **Rhode Island** | 298.27 | 2,291.51 | 298.02 | 269.92 | 436.57 | 451.13 | 4,986.56 |
| **South Carolina** | 284.42 | 2,067.34 | 280.52 | 257.40 | 406.47 | 425.59 | 4,482.46 |
| **South Dakota** | 281.06 | 2,210.63 | 278.29 | 250.68 | 412.01 | 395.02 | 4,785.57 |
| **Tennessee** | 277.06 | 2,031.03 | 271.40 | 248.62 | 395.27 | 396.74 | 4,387.75 |
| **Texas** | 292.24 | 2,191.90 | 290.78 | 264.87 | 423.69 | 443.44 | 4,765.93 |
| **Utah** | 286.74 | 2,100.54 | 283.65 | 259.80 | 411.44 | 432.31 | 4,559.42 |
| **Vermont** | 283.49 | 2,193.76 | 280.94 | 254.06 | 413.78 | 407.66 | 4,754.30 |
| **Virginia** | 286.76 | 2,183.52 | 284.67 | 258.40 | 416.99 | 423.33 | 4,739.24 |
| **Washington** | 307.91 | 2,507.62 | 310.99 | 277.66 | 461.57 | 462.83 | 5,468.05 |
| **West Virginia** | 290.90 | 1,967.70 | 286.94 | 267.36 | 407.55 | 465.31 | 4,280.70 |
| **Wisconsin** | 276.87 | 2,125.40 | 272.33 | 246.79 | 401.34 | 385.65 | 4,591.80 |
| **Wyoming** | 288.89 | 2,218.46 | 287.60 | 260.53 | 421.86 | 429.03 | 4,819.58 |

64555 = Percutaneous implantation of neurostimulator electrode array; peripheral nerve. 64575 = Open implantation of neurostimulator electrode array; peripheral nerve. 64590 = Insertion or replacement of peripheral neurostimulator pulse generator or receiver. 64553 = Percutaneous implantation of neurostimulator electrode array; cranial nerve.

**eTable 5: Dollar Difference per wRVU between Medicaid and Medicare Reimbursement for CPT Codes 64555, 64575, 64590, and 64553**

| **State** | **CPT 64555 (Facility)** | **CPT 64555 (Non-Facility)** | **CPT 64575 (Facility)** | **CPT 64590 (Facility)** | **CPT 64590 (Non-Facility)** | **CPT 64553 (Facility)** | **CPT 64553 (Non-Facility)** |
| --- | --- | --- | --- | --- | --- | --- | --- |
| **Alabama** | -21.47 | N/A | -21.76 | 3.27 | N/A | -49.42 | N/A |
| **Alaska** | N/A | 117.43 | 39.25 | 69.35 | N/A | N/A | 341.35 |
| **Arizona** | 9.19 | 11.39 | 10.39 | 9.73 | 9.43 | 6.67 | -108.25 |
| **Arkansas** | -9.56 | N/A | 51.25 | 8.66 | N/A | -28.19 | N/A |
| **California** | N/A | N/A | -8.93 | -28.28 | N/A | -70.67 | -920.26 |
| **Colorado** | -6.08 | N/A | -8.65 | -21.07 | N/A | -20.21 | N/A |
| **Connecticut** | -19.04 | -194.22 | -26.32 | -19.84 | -39.64 | -32.78 | -476.73 |
| **Delaware** | -1.04 | -7.54 | -1.27 | -1.06 | -1.70 | -1.48 | -15.44 |
| **Florida** | -17.27 | N/A | -20.30 | -27.56 | N/A | -60.41 | N/A |
| **Georgia** | -31.36 | N/A | -7.21 | N/A | N/A | N/A | N/A |
| **Hawaii** | 5.67 | N/A | 5.70 | 6.25 | N/A | -0.52 | N/A |
| **Idaho** | N/A | -86.37 | -6.44 | 14.44 | N/A | 405.77 | N/A |
| **Illinois** | -15.78 | N/A | -0.77 | 0.50 | N/A | -32.72 | N/A |
| **Indiana** | N/A | -40.53 | 2.78 | 25.11 | N/A | N/A | 0.00 |
| **Iowa** | -26.54 | N/A | 1.29 | -16.78 | N/A | -43.41 | N/A |
| **Kansas** | Excluded | Excluded | Excluded | Excluded | Excluded | Excluded | Excluded |
| **Kentucky** | -37.27 | N/A | -13.97 | -26.06 | N/A | -57.32 | N/A |
| **Louisiana** | N/A | N/A | N/A | 17.84 | N/A | N/A | N/A |
| **Maine** | -14.30 | N/A | -18.66 | -14.54 | N/A | N/A | N/A |
| **Maryland** | -28.80 | -375.14 | -7.95 | -20.32 | -30.14 | -52.78 | -811.17 |
| **Massachusetts** | -11.76 | -134.52 | -18.46 | -31.58 | -51.45 | -28.55 | -565.53 |
| **Michigan** | -19.41 | -122.86 | -25.06 | -20.51 | -30.29 | N/A | N/A |
| **Minnesota** | -11.59 | -92.71 | -15.00 | -11.66 | -19.33 | -15.23 | -188.75 |
| **Mississippi** | -1.81 | -34.26 | -3.81 | -5.03 | -7.82 | -6.78 | -69.57 |
| **Missouri** | N/A | -62.72 | 10.43 | 36.04 | N/A | -8.84 | -421.44 |
| **Montana** | 26.86 | 100.85 | 32.12 | 27.21 | 27.28 | 28.92 | 38.44 |
| **Nebraska** | -13.75 | -318.40 | 54.38 | 6.74 | 16.00 | -19.87 | -685.17 |
| **Nevada** | -23.68 | N/A | 7.83 | -19.15 | N/A | -45.68 | N/A |
| **New Hampshire** | -32.28 | N/A | -26.09 | -30.84 | N/A | N/A | N/A |
| **New Jersey** | N/A | -258.09 | -37.52 | -16.35 | N/A | N/A | -592.26 |
| **New Mexico** | N/A | 144.68 | 38.26 | 72.21 | N/A | N/A | 126.46 |
| **New York** | -44.32 | -74.60 | -43.53 | -39.86 | -46.45 | -64.16 | -512.55 |
| **North Carolina** | -29.43 | -342.40 | -15.39 | -24.01 | -35.10 | N/A | N/A |
| **North Dakota** | N/A | 7.37 | 13.36 | 38.85 | N/A | N/A | 99.49 |
| **Ohio** | -21.40 | -157.52 | -18.27 | -35.93 | -54.53 | -40.82 | -521.54 |
| **Oklahoma** | -4.48 | -61.30 | -6.67 | -4.38 | -9.76 | -5.92 | -127.42 |
| **Oregon** | -10.93 | -92.05 | -14.45 | -11.13 | -18.82 | -15.48 | -189.02 |
| **Pennsylvania** | -30.12 | N/A | -9.97 | -21.34 | N/A | N/A | N/A |
| **Rhode Island** | -45.60 | N/A | -49.66 | -37.41 | N/A | N/A | N/A |
| **South Carolina** | N/A | N/A | N/A | -12.33 | N/A | N/A | N/A |
| **South Dakota** | 0.00 | -13.77 | 0.00 | 0.00 | -2.90 | 0.00 | -28.01 |
| **Tennessee** | Excluded | Excluded | Excluded | Excluded | Excluded | Excluded | Excluded |
| **Texas** | -3.66 | -87.23 | -5.73 | -3.43 | N/A | -12.74 | N/A |
| **Utah** | N/A | -107.66 | -10.60 | 9.95 | N/A | N/A | -309.09 |
| **Vermont** | -7.59 | -56.20 | -9.65 | -7.69 | -12.31 | -10.14 | -114.14 |
| **Virginia** | -1.39 | -77.56 | -3.17 | -1.24 | -12.78 | -5.22 | -261.22 |
| **Washington** | -21.20 | -225.19 | -29.39 | -21.61 | -44.02 | -33.95 | -536.94 |
| **West Virginia** | -14.42 | -94.84 | -18.45 | -15.01 | -22.60 | -21.42 | -193.36 |
| **Wisconsin** | -22.02 | N/A | 2.80 | 4.27 | N/A | -32.48 | N/A |
| **Wyoming** | -16.55 | -77.20 | -6.19 | 1.60 | -13.15 | -41.03 | -265.16 |

Dollar difference was calculated by subtracting the Medicare reimbursement rate for a given state from the Medicaid reimbursement rate for that state. 64555 = Percutaneous implantation of neurostimulator electrode array; peripheral nerve. 64575 = Open implantation of neurostimulator electrode array; peripheral nerve. 64590 = Insertion or replacement of peripheral neurostimulator pulse generator or receiver. 64553 = Percutaneous implantation of neurostimulator electrode array; cranial nerve.

**eTable 6: Analysis of relative Medicaid reimbursement for CPT Codes 64555, 64575, 64590, and 64553 by state as a fraction of corresponding Medicare rates**

| **State** | **CPT 64555 (Facility)** | | **CPT 64555 (Non-Facility)** | | **CPT 64575 (Facility)** | | **CPT 64590 (Facility)** | | **CPT 64590 (Non-Facility)** | | **CPT 64553 (Facility)** | | **CPT 64553 (Non-Facility)** | |
| --- | --- | --- | --- | --- | --- | --- | --- | --- | --- | --- | --- | --- | --- | --- |
|  | **Baseline** | **Wage-Adjusted** | **Baseline** | **Wage-Adjusted** | **Baseline** | **Wage-Adjusted** | **Baseline** | **Wage-Adjusted** | **Baseline** | **Wage-Adjusted** | **Baseline** | **Wage-Adjusted** | **Baseline** | **Wage-Adjusted** |
| **Alabama** | 0.561 | 0.444 | N/A | N/A | 0.650 | 0.514 | 1.066 | 0.844 | N/A | N/A | 0.249 | 0.197 | N/A | N/A |
| **Alaska** | N/A | N/A | 1.271 | 1.433 | 1.465 | 1.653 | 2.002 | 2.258 | N/A | N/A | N/A | N/A | 1.393 | 1.571 |
| **Arizona** | 1.179 | 1.180 | 1.030 | 1.031 | 1.156 | 1.158 | 1.185 | 1.187 | 1.112 | 1.114 | 1.092 | 1.093 | 0.862 | 0.863 |
| **Arkansas** | 0.803 | 0.689 | N/A | N/A | 1.835 | 1.575 | 1.176 | 1.009 | N/A | N/A | 0.564 | 0.484 | N/A | N/A |
| **California** | N/A | N/A | N/A | N/A | 0.876 | 1.307 | 0.490 | 0.731 | N/A | N/A | 0.061 | 0.091 | 0.025 | 0.038 |
| **Colorado** | 0.885 | 0.927 | N/A | N/A | 0.875 | 0.917 | 0.610 | 0.639 | N/A | N/A | 0.729 | 0.763 | N/A | N/A |
| **Connecticut** | 0.653 | 0.755 | 0.543 | 0.627 | 0.635 | 0.734 | 0.649 | 0.750 | 0.567 | 0.655 | 0.596 | 0.689 | 0.453 | 0.524 |
| **Delaware** | 0.980 | 1.063 | 0.981 | 1.064 | 0.981 | 1.065 | 0.980 | 1.063 | 0.980 | 1.063 | 0.980 | 1.063 | 0.981 | 1.064 |
| **Florida** | 0.689 | 0.645 | N/A | N/A | 0.723 | 0.677 | 0.528 | 0.494 | N/A | N/A | 0.320 | 0.299 | N/A | N/A |
| **Georgia** | 0.401 | 0.339 | N/A | N/A | 0.894 | 0.755 | N/A | N/A | N/A | N/A | N/A | N/A | N/A | N/A |
| **Hawaii** | 1.107 | 1.419 | N/A | N/A | 1.082 | 1.386 | 1.116 | 1.431 | N/A | N/A | 0.993 | 1.273 | N/A | N/A |
| **Idaho** | N/A | N/A | 0.763 | 0.765 | 0.898 | 0.900 | 1.290 | 1.293 | N/A | N/A | 7.171 | 7.190 | N/A | N/A |
| **Illinois** | 0.713 | 0.721 | N/A | N/A | 0.989 | 1.000 | 1.009 | 1.020 | N/A | N/A | 0.626 | 0.633 | N/A | N/A |
| **Indiana** | N/A | N/A | 0.890 | 0.840 | 1.044 | 0.985 | 1.502 | 1.417 | N/A | N/A | N/A | N/A | 1.000 | 0.944 |
| **Iowa** | 0.457 | 0.408 | N/A | N/A | 1.021 | 0.911 | 0.660 | 0.589 | N/A | N/A | 0.327 | 0.292 | N/A | N/A |
| **Kansas** | Excluded | -- | Excluded | -- | Excluded | -- | Excluded | -- | Excluded | -- | Excluded | -- | Excluded | -- |
| **Kentucky** | 0.259 | 0.233 | N/A | N/A | 0.783 | 0.706 | 0.495 | 0.446 | N/A | N/A | 0.194 | 0.175 | N/A | N/A |
| **Louisiana** | N/A | N/A | N/A | N/A | N/A | N/A | 1.339 | 1.125 | N/A | N/A | N/A | N/A | N/A | N/A |
| **Maine** | 0.716 | 0.685 | N/A | N/A | 0.712 | 0.681 | 0.716 | 0.685 | N/A | N/A | N/A | N/A | N/A | N/A |
| **Maryland** | 0.463 | 0.442 | 0.089 | 0.084 | 0.887 | 0.846 | 0.632 | 0.602 | 0.661 | 0.631 | 0.324 | 0.309 | 0.038 | 0.037 |
| **Massachusetts** | 0.785 | 0.880 | 0.695 | 0.779 | 0.743 | 0.834 | 0.434 | 0.487 | 0.442 | 0.496 | 0.632 | 0.709 | 0.373 | 0.419 |
| **Michigan** | 0.632 | 0.575 | 0.673 | 0.612 | 0.634 | 0.577 | 0.625 | 0.569 | 0.645 | 0.586 | N/A | N/A | N/A | N/A |
| **Minnesota** | 0.770 | 0.806 | 0.770 | 0.806 | 0.770 | 0.806 | 0.770 | 0.806 | 0.770 | 0.806 | 0.770 | 0.806 | 0.770 | 0.806 |
| **Mississippi** | 0.963 | 0.768 | 0.901 | 0.718 | 0.939 | 0.749 | 0.900 | 0.717 | 0.900 | 0.717 | 0.900 | 0.717 | 0.901 | 0.718 |
| **Missouri** | N/A | N/A | 0.829 | 0.744 | 1.159 | 1.040 | 1.688 | 1.516 | N/A | N/A | 0.879 | 0.789 | 0.436 | 0.391 |
| **Montana** | 1.514 | 1.524 | 1.255 | 1.264 | 1.472 | 1.482 | 1.507 | 1.517 | 1.317 | 1.326 | 1.384 | 1.393 | 1.047 | 1.055 |
| **Nebraska** | 0.719 | 0.687 | 0.130 | 0.124 | 1.870 | 1.786 | 1.136 | 1.085 | 1.201 | 1.148 | 0.692 | 0.661 | 0.077 | 0.073 |
| **Nevada** | 0.542 | 0.582 | N/A | N/A | 1.116 | 1.197 | 0.638 | 0.684 | N/A | N/A | 0.375 | 0.402 | N/A | N/A |
| **New Hampshire** | 0.385 | 0.421 | N/A | N/A | 0.620 | 0.677 | 0.426 | 0.465 | N/A | N/A | N/A | N/A | N/A | N/A |
| **New Jersey** | N/A | N/A | 0.417 | 0.478 | 0.493 | 0.565 | 0.717 | 0.822 | N/A | N/A | N/A | N/A | 0.348 | 0.399 |
| **New Mexico** | N/A | N/A | 1.395 | 1.295 | 1.573 | 1.461 | 2.352 | 2.184 | N/A | N/A | N/A | N/A | 1.169 | 1.085 |
| **New York** | 0.221 | 0.265 | 0.830 | 0.995 | 0.420 | 0.503 | 0.322 | 0.386 | 0.511 | 0.613 | 0.248 | 0.297 | 0.433 | 0.519 |
| **North Carolina** | 0.412 | 0.374 | 0.075 | 0.069 | 0.760 | 0.692 | 0.528 | 0.480 | 0.568 | 0.516 | N/A | N/A | N/A | N/A |
| **North Dakota** | N/A | N/A | 1.019 | 1.019 | 1.206 | 1.206 | 1.765 | 1.765 | N/A | N/A | N/A | N/A | 1.124 | 1.124 |
| **Ohio** | 0.580 | 0.524 | 0.568 | 0.513 | 0.722 | 0.652 | 0.314 | 0.284 | 0.335 | 0.303 | 0.441 | 0.399 | 0.298 | 0.270 |
| **Oklahoma** | 0.910 | 0.791 | 0.828 | 0.719 | 0.895 | 0.778 | 0.914 | 0.794 | 0.878 | 0.763 | 0.915 | 0.795 | 0.824 | 0.716 |
| **Oregon** | 0.792 | 0.948 | 0.778 | 0.931 | 0.789 | 0.945 | 0.792 | 0.948 | 0.785 | 0.940 | 0.789 | 0.945 | 0.777 | 0.930 |
| **Pennsylvania** | 0.427 | 0.421 | N/A | N/A | 0.854 | 0.842 | 0.605 | 0.596 | N/A | N/A | N/A | N/A | N/A | N/A |
| **Rhode Island** | 0.141 | 0.159 | N/A | N/A | 0.282 | 0.319 | 0.311 | 0.352 | N/A | N/A | N/A | N/A | N/A | N/A |
| **South Carolina** | N/A | N/A | N/A | N/A | N/A | N/A | 0.762 | 0.660 | N/A | N/A | N/A | N/A | N/A | N/A |
| **South Dakota** | 1.000 | 1.000 | 0.965 | 0.965 | 1.000 | 1.000 | 1.000 | 1.000 | 0.965 | 0.965 | 1.000 | 1.000 | 0.965 | 0.965 |
| **Tennessee** | Excluded | -- | Excluded | -- | Excluded | -- | Excluded | -- | Excluded | -- | Excluded | -- | Excluded | -- |
| **Texas** | 0.930 | 0.858 | 0.776 | 0.717 | 0.915 | 0.845 | 0.936 | 0.864 | N/A | N/A | 0.828 | 0.764 | N/A | N/A |
| **Utah** | N/A | N/A | 0.712 | 0.686 | 0.839 | 0.808 | 1.190 | 1.146 | N/A | N/A | N/A | N/A | 0.595 | 0.573 |
| **Vermont** | 0.849 | 0.871 | 0.856 | 0.878 | 0.852 | 0.874 | 0.850 | 0.871 | 0.852 | 0.874 | 0.851 | 0.873 | 0.856 | 0.878 |
| **Virginia** | 0.973 | 0.890 | 0.800 | 0.732 | 0.952 | 0.871 | 0.976 | 0.893 | 0.848 | 0.775 | 0.926 | 0.847 | 0.670 | 0.613 |
| **Washington** | 0.613 | 0.707 | 0.495 | 0.571 | 0.593 | 0.683 | 0.613 | 0.707 | 0.526 | 0.607 | 0.561 | 0.647 | 0.413 | 0.476 |
| **West Virginia** | 0.721 | 0.597 | 0.729 | 0.604 | 0.723 | 0.598 | 0.721 | 0.597 | 0.724 | 0.600 | 0.725 | 0.600 | 0.730 | 0.604 |
| **Wisconsin** | 0.553 | 0.536 | N/A | N/A | 1.044 | 1.011 | 1.086 | 1.051 | N/A | N/A | 0.496 | 0.481 | N/A | N/A |
| **Wyoming** | 0.678 | 0.678 | 0.804 | 0.804 | 0.907 | 0.907 | 1.030 | 1.030 | 0.845 | 0.845 | 0.428 | 0.428 | 0.671 | 0.671 |

Percentages were calculated by dividing the Medicaid reimbursement rate by the Medicare reimbursement rate for a given state, then multiplying by 100 to express as a percentage. 64555 = Percutaneous implantation of neurostimulator electrode array; peripheral nerve. 64575 = Open implantation of neurostimulator electrode array; peripheral nerve. 64590 = Insertion or replacement of peripheral neurostimulator pulse generator or receiver. 64553 = Percutaneous implantation of neurostimulator electrode array; cranial nerve.

**eTable 7: Medicare Service Utilization, Rendering Providers, Beneficiaries, and Average Standardized Payment for CPT Code 64555 Between 2013 and 2024**

| **Year** | **Total Rendering Providers (Facility)** | **Total Beneficiaries (Facility)** | **Total Services (Facility)** | **Avg Medicare Standardized Payment (Facility) ($)** | **Total Rendering Providers (Non-Facility)** | **Total Beneficiaries (Non-Facility)** | **Total Services (Non-Facility)** | **Avg Medicare Standardized Payment (Non-Facility) ($)** |
| --- | --- | --- | --- | --- | --- | --- | --- | --- |
| **2013** | 586 | 2,383 | 8,705 | 1,349.92 | 670 | 4,362 | 9,889 | 156.50 |
| **2014** | 514 | 2,279 | 8,982 | 1,369.13 | 565 | 3,344 | 7,837 | 147.91 |
| **2015** | 444 | 2,055 | 7,956 | 1,553.36 | 306 | 1,124 | 4,376 | 144.54 |
| **2016** | 409 | 2,080 | 8,440 | 1,641.10 | 261 | 1,103 | 3,772 | 137.28 |
| **2017** | 317 | 837 | 2,848 | 1,562.46 | 156 | 691 | 2,578 | 116.91 |
| **2018** | 394 | 750 | 1,961 | 1,511.65 | 168 | 615 | 937 | 810.01 |
| **2019** | 702 | 1,427 | 4,409 | 1,629.85 | 246 | 848 | 1,203 | 884.73 |
| **2020** | 872 | 2,051 | 6,591 | 1,756.00 | 344 | 1,308 | 1,768 | 1,060.42 |
| **2021** | 1,238 | 3,246 | 9,747 | 1,671.33 | 425 | 1,619 | 2,249 | 1,222.90 |
| **2022** | 1,582 | 3,963 | 11,983 | 1,676.56 | 489 | 1,802 | 2,589 | 1,171.15 |
| **2023** | 2,034 | 5,472 | 16,990 | 1,937.16 | 565 | 1,945 | 2,981 | 1,041.65 |
| **2024** | 2,394 | 6,888 | 22,494 | 2,005.60 | 664 | 2,391 | 3,663 | 1,010.64 |

“Total services” refers to the number of procedures billed under a given CPT/HCPCS code in a given year. “Total rendering providers” refers to the number of unique providers (NPIs) who performed at least one service for a CPT/HCPCS code in a given year. “Total beneficiaries” refers to the number of unique Medicare patients who received a service in a given calendar year. “Average Medicare standardized payment” is defined by CMS as the average amount that Medicare paid after beneficiary deductible and coinsurance amounts have been deducted for the line item service and after standardization of the Medicare payment has been applied. Standardization removes geographic differences in payment rates for individual services, such as those that account for local wages or input prices and makes Medicare payments across geographic areas comparable, so that differences reflect variation in factors such as physicians’ practice patterns and beneficiaries’ ability and willingness to obtain care. 64555 = Percutaneous implantation of neurostimulator electrode array; peripheral nerve. 64575 = Open implantation of neurostimulator electrode array; peripheral nerve. 64590 = Insertion or replacement of peripheral neurostimulator pulse generator or receiver.

**eTable 8: Medicare Service Utilization, Rendering Providers, Beneficiaries, and Average Standardized Payment for CPT Code 64590 Between 2013 and 2024**

| **Year** | **Total Rendering Providers (Facility)** | **Total Beneficiaries (Facility)** | **Total Services (Facility)** | **Avg Medicare Standardized Payment (Facility) ($)** | **Total Rendering Providers (Non-Facility)** | **Total Beneficiaries (Non-Facility)** | **Total Services (Non-Facility)** | **Avg Medicare Standardized Payment (Non-Facility) ($)** |
| --- | --- | --- | --- | --- | --- | --- | --- | --- |
| **2013** | 3,092 | 10,677 | 13,154 | 2,374.58 | 66 | 90 | 91 | 158.78 |
| **2014** | 3,167 | 11,059 | 13,937 | 2,657.57 | 74 | 105 | 110 | 156.93 |
| **2015** | 3,129 | 10,577 | 13,185 | 2,601.81 | 67 | 94 | 96 | 145.88 |
| **2016** | 3,199 | 11,681 | 14,823 | 2,561.23 | 137 | 316 | 322 | 114.07 |
| **2017** | 3,143 | 11,502 | 14,699 | 2,811.35 | 125 | 314 | 319 | 114.34 |
| **2018** | 3,161 | 11,468 | 14,748 | 2,922.54 | 108 | 249 | 251 | 109.37 |
| **2019** | 3,389 | 12,147 | 15,659 | 3,107.13 | 127 | 353 | 364 | 107.85 |
| **2020** | 3,414 | 11,614 | 15,625 | 3,667.16 | 132 | 366 | 373 | 98.85 |
| **2021** | 3,855 | 14,880 | 20,036 | 3,808.22 | 150 | 445 | 448 | 101.63 |
| **2022** | 4,132 | 15,693 | 21,278 | 3,907.32 | 154 | 394 | 397 | 99.16 |
| **2023** | 4,576 | 17,749 | 24,526 | 4,317.68 | 190 | 509 | 529 | 99.98 |
| **2024** | 5,055 | 18,547 | 26,152 | 5,462.07 | 217 | 552 | 562 | 173.18 |

“Total services” refers to the number of procedures billed under a given CPT/HCPCS code in a given year. “Total rendering providers” refers to the number of unique providers (NPIs) who performed at least one service for a CPT/HCPCS code in a given year. “Total beneficiaries” refers to the number of unique Medicare patients who received a service in a given calendar year. “Average Medicare standardized payment” is defined by CMS as the average amount that Medicare paid after beneficiary deductible and coinsurance amounts have been deducted for the line item service and after standardization of the Medicare payment has been applied. Standardization removes geographic differences in payment rates for individual services, such as those that account for local wages or input prices and makes Medicare payments across geographic areas comparable, so that differences reflect variation in factors such as physicians’ practice patterns and beneficiaries’ ability and willingness to obtain care. 64555 = Percutaneous implantation of neurostimulator electrode array; peripheral nerve. 64575 = Open implantation of neurostimulator electrode array; peripheral nerve. 64590 = Insertion or replacement of peripheral neurostimulator pulse generator or receiver.

**eTable 9: Medicare Service Utilization, Rendering Providers, Beneficiaries, and Average Standardized Payment for CPT Code 64575 Between 2013 and 2024**

| **Year** | **Total Rendering Providers (Facility)** | **Total Beneficiaries (Facility)** | **Total Services (Facility)** | **Avg Medicare Standardized Payment (Facility) ($)** | **Total Rendering Providers (Non-Facility)** | **Total Beneficiaries (Non-Facility)** | **Total Services (Non-Facility)** | **Avg Medicare Standardized Payment (Non-Facility) ($)** |
| --- | --- | --- | --- | --- | --- | --- | --- | --- |
| **2013** | 262 | 611 | 1,323 | 1,029.75 | 18 | 69 | 128 | 189.02 |
| **2014** | 211 | 490 | 1,069 | 1,173.03 | 15 | 50 | 95 | 139.41 |
| **2015** | 165 | 378 | 729 | 778.89 | 14 | 19 | 28 | 177.03 |
| **2016** | 127 | 279 | 595 | 896.55 | Not reported | Not reported | Not reported | Not reported |
| **2017** | 128 | 238 | 524 | 3,378.19 | Not reported | Not reported | Not reported | Not reported |
| **2018** | 138 | 229 | 480 | 3,340.47 | 11 | 13 | 18 | 190.31 |
| **2019** | 150 | 233 | 514 | 4,346.91 | 13 | 19 | 26 | 203.43 |
| **2020** | 159 | 268 | 610 | 4,918.59 | Not reported | Not reported | Not reported | Not reported |
| **2021** | 186 | 327 | 730 | 3,363.02 | 13 | 18 | 24 | 199.49 |
| **2022** | 128 | 248 | 630 | 3,683.02 | Not reported | Not reported | Not reported | Not reported |
| **2023** | 127 | 227 | 530 | 3,089.72 | Not reported | Not reported | Not reported | Not reported |
| **2024** | 90 | 144 | 316 | 3,448.72 | Not reported | Not reported | Not reported | Not reported |

“Total services” refers to the number of procedures billed under a given CPT/HCPCS code in a given year. “Total rendering providers” refers to the number of unique providers (NPIs) who performed at least one service for a CPT/HCPCS code in a given year. “Total beneficiaries” refers to the number of unique Medicare patients who received a service in a given calendar year. “Average Medicare standardized payment” is defined by CMS as the average amount that Medicare paid after beneficiary deductible and coinsurance amounts have been deducted for the line item service and after standardization of the Medicare payment has been applied. Standardization removes geographic differences in payment rates for individual services, such as those that account for local wages or input prices and makes Medicare payments across geographic areas comparable, so that differences reflect variation in factors such as physicians’ practice patterns and beneficiaries’ ability and willingness to obtain care. 64575 = Open implantation of neurostimulator electrode array; peripheral nerve.

**eTable 10: Medicare Service Utilization, Rendering Providers, Beneficiaries, and Average Standardized Payment for CPT Code 64553 Between 2013 and 2024**

| **Year** | **Total Rendering Providers (Facility)** | **Total Beneficiaries (Facility)** | **Total Services (Facility)** | **Avg Medicare Standardized Payment (Facility) ($)** | **Total Rendering Providers (Non-Facility)** | **Total Beneficiaries (Non-Facility)** | **Total Services (Non-Facility)** | **Avg Medicare Standardized Payment (Non-Facility) ($)** |
| --- | --- | --- | --- | --- | --- | --- | --- | --- |
| **2013** | 62 | 287 | 1,072 | 1,392.88 | 43 | 574 | 1,048 | 186.35 |
| **2014** | 36 | 114 | 333 | 1,173.00 | 30 | 133 | 330 | 164.97 |
| **2015** | 28 | 71 | 374 | 1,451.22 | 10 | 17 | 42 | 164.74 |
| **2016** | Not reported | Not reported | Not reported | Not reported | 10 | 21 | 24 | 148.40 |
| **2017** | 25 | 60 | 99 | 1,592.62 | 49 | 689 | 1,404 | 145.80 |
| **2018** | 33 | 51 | 65 | 851.70 | 59 | 565 | 913 | 826.84 |
| **2019** | 28 | 49 | 82 | 837.39 | 14 | 247 | 478 | 1,340.31 |
| **2020** | 29 | 54 | 75 | 876.69 | 5 | 100 | 138 | 1,610.92 |
| **2021** | 29 | 82 | 153 | 882.91 | 3 | 49 | 56 | 1,952.52 |
| **2022** | 21 | 60 | 74 | 3,481.86 | Not reported | Not reported | Not reported | Not reported |
| **2023** | 21 | 26 | 38 | 1,829.33 | Not reported | Not reported | Not reported | Not reported |
| **2024** | 17 | 47 | 63 | 4,426.07 | Not reported | Not reported | Not reported | Not reported |

“Total services” refers to the number of procedures billed under a given CPT/HCPCS code in a given year. “Total rendering providers” refers to the number of unique providers (NPIs) who performed at least one service for a CPT/HCPCS code in a given year. “Total beneficiaries” refers to the number of unique Medicare patients who received a service in a given calendar year. “Average Medicare standardized payment” is defined by CMS as the average amount that Medicare paid after beneficiary deductible and coinsurance amounts have been deducted for the line item service and after standardization of the Medicare payment has been applied. Standardization removes geographic differences in payment rates for individual services, such as those that account for local wages or input prices and makes Medicare payments across geographic areas comparable, so that differences reflect variation in factors such as physicians’ practice patterns and beneficiaries’ ability and willingness to obtain care. 64553 = Percutaneous implantation of neurostimulator electrode array; cranial nerve.

**eFigure 1: Wage-Adjusted Medicaid Reimbursement Rates for CPT Codes 64555, 64575, 64590, and 64553 (Facility)**

A heatmap of the United States representing wage-adjusted Medicaid reimbursement rate by state for CPT codes 64555 (A), 64575 (B), 64590 (C), and 64553 (D). Orange shades represent states with wage-adjusted Medicaid reimbursement rates above the median value of all states included in the analysis for a given CPT code, while blue shades represent states with wage-adjusted Medicaid reimbursement rates below the median value of all states included in the analysis for a given CPT code. States shaded gray were excluded from this analysis or lacked a facility setting reported rate for a given CPT code (see Methods).

**eFigure 2: Wage-Adjusted Medicaid Reimbursement Rates for CPT Codes 64555, 64590, and 64553 (Non-Facility)**

A heatmap of the United States representing wage-adjusted Medicaid reimbursement rate by state for CPT codes 64555 (A), 64590 (B), and 64553 (C) in non-facility settings. Orange shades represent states with wage-adjusted Medicaid reimbursement rates above the median value of all states included in the analysis for a given CPT code, while blue shades represent states with wage-adjusted Medicaid reimbursement rates below the median value of all states included in the analysis for a given CPT code. States shaded gray were excluded from this analysis or lacked a non-facility setting reported rate for a given CPT code (see Methods).

**eFigure 3: Medicare Reimbursement Rates for CPT Codes 64555, 64575, 64590, and 64553 (Facility)**

A heatmap of the United States representing Medicare reimbursement rate by state for CPT codes 64555 (A), 64575 (B), 64590 (C), and 64553 (D) in facility settings. Orange shades represent states with Medicaid reimbursement rates above the median value of all states included in the analysis for a given CPT code, while blue shades represent states with Medicaid reimbursement rates below the median value of all states included in the analysis for a given CPT code.

**eFigure 4: Medicare Reimbursement Rates for CPT Codes 64555, 64590, and 64553 (Non-Facility)**

A heatmap of the United States representing Medicare reimbursement rate by state for CPT codes 64555 (A), 64590 (B), and 64553 (C) in non-facility settings. Orange shades represent states with Medicare reimbursement rates above the median value of all states included in the analysis for a given CPT code, while blue shades represent states with Medicare reimbursement rates below the median value of all states included in the analysis for a given CPT code.
